# Bacterially induced synthesis of low-Cd struvite coupled with Cd^2+^ biosorption under high cadmium stress

**DOI:** 10.1039/d6ra05024g

**Published:** 2026-09-21

**Authors:** Lei Meng, Bin Lian

**Affiliations:** a College of Life Science, Nanjing Normal University Nanjing 210023 China; b Jiangsu Key Laboratory of Ocean-Land Environmental Change and Ecological Construction, College of Marine Science and Engineering, Nanjing Normal University Nanjing 210023 China bin2368@vip.163.com

## Abstract

Chemical precipitation of struvite is promising for phosphorus recovery from wastewater. However, metal cations with high phosphate affinity, such as Cd^2+^, can reduce struvite phase purity and promote heavy-metal incorporation, limiting practical safety. In this study, seawater was used as the magnesium source and *Shewanella oneidensis* MR-1 as the mineralizing strain to establish a struvite biomineralization system under Cd^2+^ stress, aiming to remove aqueous Cd^2+^ using MR-1 while producing struvite with low Cd content. An artificial Cd^2+^ stress gradient of 1–30 mg L^−1^ was applied, with 30 mg L^−1^ representing an extreme high-Cd condition. The results showed that MR-1 induced the formation of struvite-dominated precipitates across the tested Cd^2+^ concentration range, with calculated struvite contents of 96.03–97.36%. MR-1 also removed 85.16–100% of Cd^2+^ from the aqueous phase through biosorption. At the same initial Cd^2+^ concentrations, bio-struvite contained 7.13–354.35 mg kg^−1^ Cd, significantly lower than the 923.74–18339.83 mg kg^−1^ measured in chemically precipitated struvite. Total phosphorus removal efficiencies were 88.27–91.46% and 58.12–60.23% in the biomineralization and chemical precipitation systems, respectively. TEM-EDS and FTIR analyses indicated that carboxyl, hydroxyl, amino, amide, and phosphorus-containing functional groups on the MR-1 surface were involved in Cd^2+^ immobilization. The good fit to the pseudo-second-order model further supported an important role of chemisorption in Cd^2+^ immobilization. This study provides a new strategy for the resource-oriented treatment of Cd-contaminated eutrophic wastewater.

## Abstract 2

*Shewanella oneidensis* MR-1 coupled struvite recovery with Cd^2+^ biosorption, reducing Cd incorporation into the recovered mineral while maintaining high TP removal.

## Graphical abstract

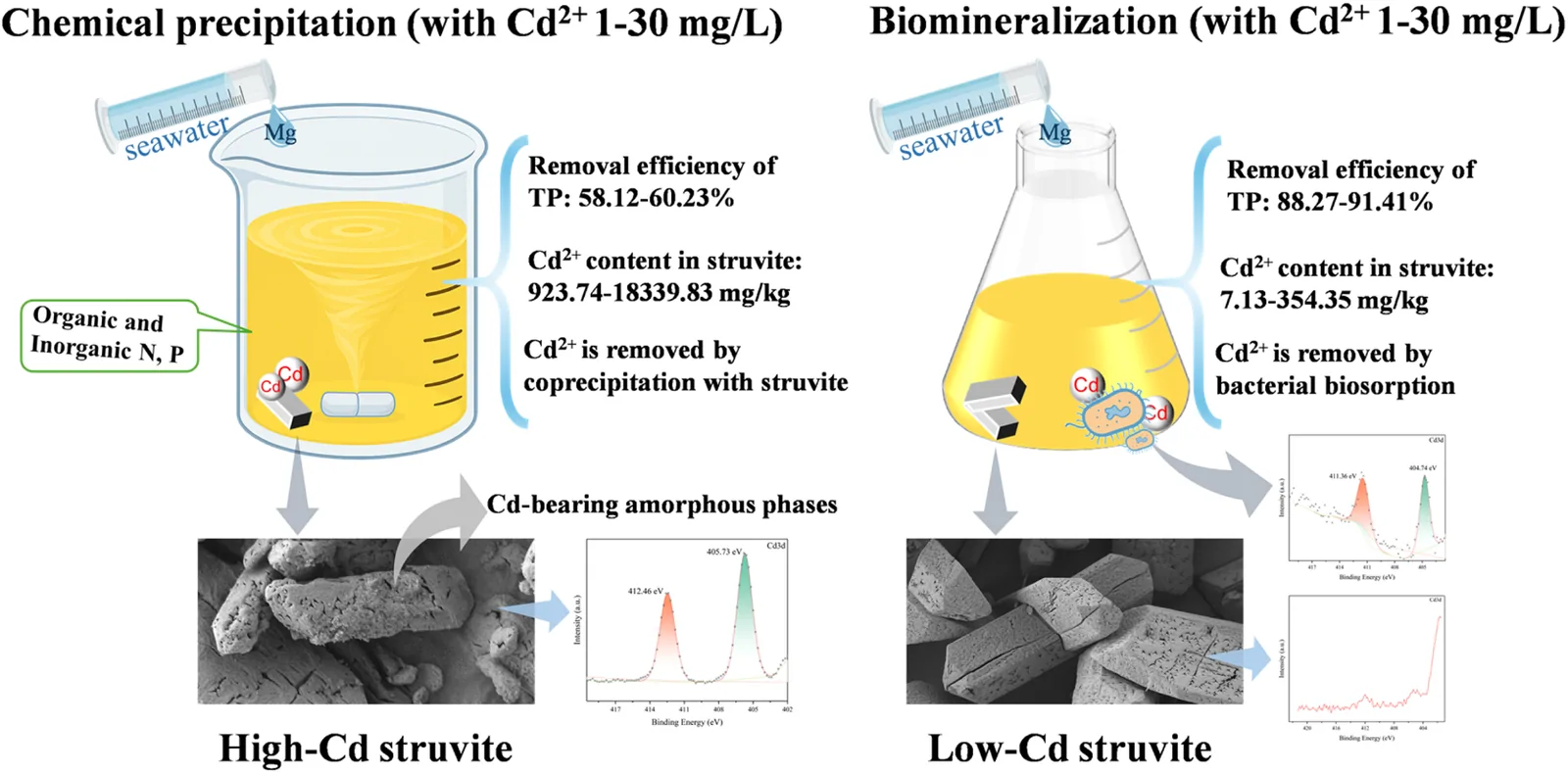

## 1. Introduction

In recent decades, human activities have profoundly altered the global phosphorus cycle.^1^ Large amounts of nitrogen and phosphorus enter municipal, agricultural, and industrial wastewater, making simultaneous pollution control and nutrient recovery an important direction in wastewater treatment.^2,3^ Current nitrogen and phosphorus recovery technologies mainly include adsorption, ion exchange, chemical crystallization, membrane separation, and biological or integrated processes.^4^ Adsorption and ion exchange offer operational simplicity and tunable selectivity, but their applications are constrained by adsorption capacity, material regeneration, and operating costs.^5,6^ Membrane processes enable nutrient concentration and selective separation, but membrane fouling and energy consumption remain major barriers to large-scale application.^7^ Crystallization can directly yield recoverable mineral products, but its efficiency and product composition are readily affected by pH, ion competition, and complex water chemistry.^8^ Biological and integrated technologies can enhance the recovery potential of different forms of nitrogen and phosphorus by coupling microbial enrichment, nutrient transformation, and downstream recovery processes.^9^ Therefore, achieving efficient nutrient recovery, synergistic contaminant removal, and product safety under complex water–matrix conditions remains a key challenge in nitrogen and phosphorus resource recovery.

Among phosphorus recovery strategies, chemical precipitation of struvite (MgNH_4_PO_4_·6H_2_O) is particularly attractive because of its mild reaction conditions and the potential use of the recovered product as a slow-release fertilizer.^8,10,11^ This process generally involves adding a magnesium source to wastewater containing ammonium and phosphate, followed by adjustment of the pH to an alkaline range to induce struvite precipitation.^12^ However, wastewater often has a complex composition and may contain heavy-metal ions such as Cd^2+^, Cu^2+^, Zn^2+^, Ni^2+^, and Pb^2+^, which can interfere with struvite crystallization.^12,13^ Among these ions, Cd^2+^ has a strong affinity for phosphate and can compete with Mg^2+^ during nucleation and crystal growth, forming Cd–P- or Cd–OH-related species and thereby increasing the risk of Cd transfer to the solid phase.^14,15^ Previous studies have reported that even when Cd^2+^ concentrations in some phosphorus-rich wastewaters are below 1 mg L^−1^, chemically precipitated struvite may still contain relatively high levels of Cd, far exceeding the 10 mg kg^−1^ limit specified in the Chinese standard for toxic and hazardous substances in fertilizers (GB 38400-2019).^14,16^ Therefore, promoting struvite formation while minimizing Cd incorporation into the recovered mineral is critical for the subsequent resource recovery from Cd-containing wastewater.

Microbially induced struvite mineralization provides a new route for reducing the interference of heavy metals with phosphorus recovery.^17,18^ Microbial cell surfaces and extracellular polymeric substances (EPS) can not only provide mineral nucleation sites and regulate the local ionic environment,^19–22^ but their carboxyl, hydroxyl, and phosphorus-containing functional groups can also immobilize Cd^2+^ through complexation or ion exchange, thereby reducing the likelihood of Cd^2+^ participating in struvite nucleation and growth.^23–25^*Shewanella oneidensis* MR-1 has a strong capacity to induce mineral formation and can immobilize Cd^2+^ through binding sites on the cell surface, thereby reducing its mobility and bioavailability.^26,27^ In addition, MR-1 can promote the conversion of organic phosphorus into mineralizable inorganic phosphorus through extracellular enzymatic processes, providing potential for struvite mineralization under Cd stress.^28^ On the other hand, the use of commercial magnesium salts such as MgCl_2_ and MgSO_4_ increases the cost of struvite recovery, whereas Mg^2+^-rich natural seawater may serve as a potential low-cost magnesium source.^22^ Natural seawater has a complex ionic composition, and components such as Ca^2+^, Na^+^, K^+^, Cl^−^, and SO_4_^2−^, together with its relatively high ionic strength, may affect bacterial physiological responses, EPS secretion, and struvite nucleation and crystal growth, thereby altering the overall mineralization process. Therefore, struvite biomineralization using natural seawater as the magnesium source is governed not by Mg^2+^ alone but by microbial metabolism, the complex ionic environment, and mineral formation. The effects of seawater on MR-1 mineralization behavior and Cd^2+^ partitioning therefore require further investigation.

In this study, natural seawater was used as a low-cost magnesium source to establish an *S. oneidensis* MR-1-mediated struvite biomineralization system and to evaluate its feasibility for promoting struvite formation while simultaneously removing aqueous Cd^2+^ under Cd stress. A Cd^2+^ stress gradient of 1–30 mg L^−1^ was established to investigate the ability of MR-1 to maintain struvite mineralization at different Cd^2+^ levels, the partitioning of Cd among the aqueous, cellular, and mineral phases, and the associated immobilization mechanisms, with 30 mg L^−1^ representing an extreme high-Cd stress condition. An abiotic chemical precipitation system was also included as a reference to compare Cd solid-phase partitioning and total phosphorus removal characteristics under the respective reaction conditions. This study provides an experimental basis for coupling phosphorus recovery with Cd^2+^ control in Cd-contaminated, phosphorus-rich wastewater and informs the further application of this biomineralization strategy to appropriately pretreated industrial phosphorus-containing wastewater.

## 2. Materials and methods

### 2.1. Experimental strain and cultivation

*S*. *oneidensis* MR-1 was obtained from the China Center for Type Culture Collection (CCTCC AB 2013238). After activation, the strain was cultured in sterile Luria–Bertani (LB) medium containing 1.0 g tryptone, 0.5 g yeast extract, 1.0 g NaCl, and 100 mL distilled water under shaking conditions at 30 °C and 180 rpm for 14 h until it reached the logarithmic growth phase. The resulting culture, with a cell density of approximately 4.5 × 10^9^ CFU mL^−1^, was used as the bacterial seed solution for subsequent experiments.

### 2.2. Biomineralization of struvite under Cd^2+^ stress

A Cd^2+^ stock solution (4.0 g L^−1^) was prepared using analytical-grade CdCl_2_·2.5H_2_O and sterilized by filtration through a sterile 0.22 µm cellulose acetate membrane. Appropriate volumes of the Cd^2+^ stock solution were added to 100 mL of sterile biomineralization medium containing 1.0 g tryptone, 0.5 g yeast extract, 1.0 g NaCl, 90.0 mL distilled water, and 10.0 mL seawater to establish the experimental group, hereafter referred to as the biomineralization system (BIS). The basic chemical composition of the seawater used in this study is provided in Table S1. The control group (CK) was prepared using LB medium without seawater. The Cd^2+^ concentrations in both media were adjusted to 0, 1, 10, 20, and 30 mg L^−1^. The 1–30 mg L^−1^ Cd^2+^ series was designed as an experimental stress gradient rather than to simulate Cd concentrations typical of eutrophic waters, with 1–20 mg L^−1^ representing low-to-moderate Cd stress and 30 mg L^−1^ representing an extreme high-Cd stress condition. Subsequently, 1.0 mL of the bacterial seed culture (see Section 2.1) was inoculated into each medium. All cultures were incubated in a shaker at 30 °C and 130 rpm for 6 days. After incubation, the cultures were left undisturbed on the laboratory bench for 30 min to allow natural sedimentation. The precipitates deposited at the bottom of the flasks were collected using a pipette. The remaining bacterial suspensions were centrifuged at 11 200×*g* for 10 min to separate the supernatant from bacterial cells. The collected precipitates were thoroughly washed with distilled water and anhydrous ethanol, oven-dried at 50 °C, ground using an agate mortar, and passed through a 100-mesh sieve for subsequent analysis. All experiments were performed in triplicate.

### 2.3. Chemical precipitation of struvite in the presence of Cd^2+^

For comparison, chemical precipitation of struvite was conducted in parallel. The chemical precipitation system (CHS) was prepared in a final volume of 100 mL, with a composition corresponding to that of the biomineralization medium described in Section 2.2, supplemented with appropriate amounts of ammonium salt and Cd^2+^ stock solution. The ammonium salt was added to match the maximum NH_4_^+^ concentration observed in the biomineralization system before crystal formation, which was approximately 57.70 mmol L^−1^ according to continuous sampling during the biomineralization process (Fig. S1). Accordingly, 0.3086 g of NH_4_Cl was added to the system. The pH was then adjusted to 9.10 using 1 mol L^−1^ NaOH to match the peak pH recorded in the biomineralization system (Fig. S1). After reaction for 30 min under constant stirring, precipitates were collected as described in Section 2.2. All experiments were conducted in triplicate.

### 2.4. Removal of Cd^2+^ from aqueous solution by MR-1

To elucidate the mechanism of Cd^2+^ removal by MR-1, biosorption experiments were performed. First, MR-1 was cultured in LB medium at 30 °C and 180 rpm for 24 h. The cells were harvested by centrifugation at 11 200×*g* for 10 min and washed three times with sterile distilled water. The collected biomass was frozen at −80 °C for 2–3 h and then lyophilized at −58 °C for 24 h using a vacuum freeze-dryer (Alpha 1-2 LDplus, Martin Christ, Germany). After lyophilization, the biomass was ground and passed through a 100-mesh sieve to obtain the biosorbent for subsequent experiments.^24^

The effects of the initial Cd^2+^ concentration (10–60 mg L^−1^, pH = 6.0) and contact time (5–720 min) on Cd^2+^ biosorption were evaluated at 30 °C and 130 rpm with a biosorbent dosage of 0.5 g L^−1^. The biosorption capacities at time *t* (*q*_*t*_) and at equilibrium (*q*_e_) were calculated using eqn (1):^29^1
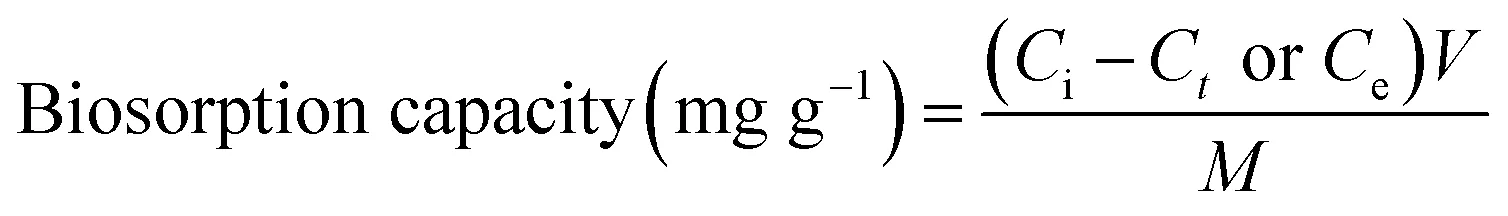
where *C*_i_, *C*_*t*_, and *C*_e_ represent the initial Cd^2+^ concentration, the Cd^2+^ concentration at time *t*, and the equilibrium Cd^2+^ concentration (mg L^−1^), respectively; *M* is the dry weight of the biosorbent (g); and *V* is the volume of the heavy-metal solution (L).

To elucidate the Cd^2+^ biosorption mechanism of the MR-1 biosorbent, the experimental data were fitted using the Langmuir (eqn (2)) and Freundlich (eqn (3)) adsorption isotherm models. In addition, the pseudo-first-order (eqn (4)) and pseudo-second-order (eqn (5)) kinetic models were used to analyze the adsorption kinetics.^30^2
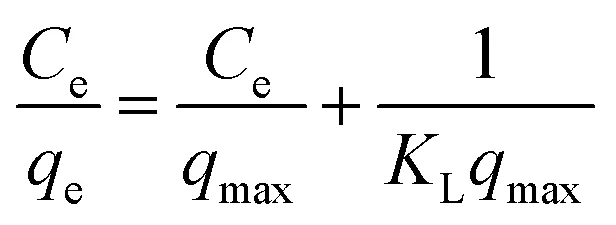
3
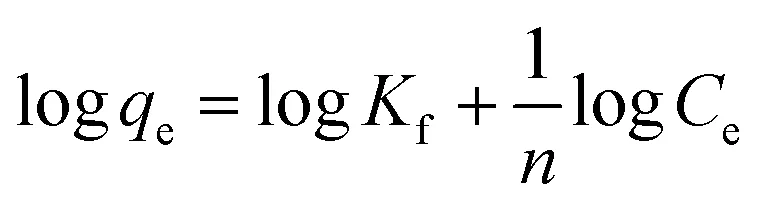
where *C*_e_ represents the equilibrium Cd^2+^ concentration in solution (mg L^−1^), *q*_e_ is the equilibrium biosorption capacity (mg g^−1^), *q*_max_ denotes the maximum monolayer biosorption capacity predicted by the Langmuir model (mg g^−1^), *K*_L_ is the Langmuir constant (L mg^−1^), and *K*_f_ and 1/*n* are the Freundlich constants.4
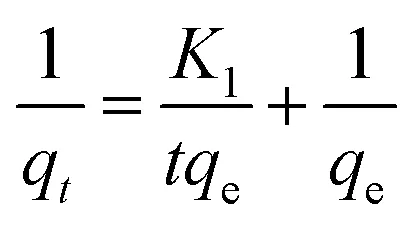
5
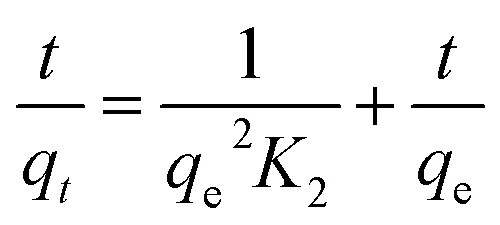


In the kinetic models, *q*_*t*_ represents the biosorption capacity at time *t* (*t* ≠ 0) (mg g^−1^), *q*_e_ is the equilibrium biosorption capacity (mg g^−1^), *t* is the adsorption time (min), *K*_1_ is the rate constant of the pseudo-first-order kinetic model (min^−1^), and *K*_2_ is the rate constant of the pseudo-second-order kinetic model (g mg^−1^ min^−1^).

### 2.5. Analytical indicators and methods

#### 2.5.1. Solution-phase analysis

The pH, bacterial growth, alkaline phosphatase (ALP) activity, and concentrations of Mg^2+^, Cd^2+^, NH_4_^+^, and total phosphorus (TP) were determined by periodically collecting culture medium samples under different experimental conditions. The pH was measured using a pH meter (PHSJ-4F, China). Bacterial growth was characterized by measuring the optical density of the bacterial suspension at 600 nm (OD_600_) using a spectrophotometer (V-5000, China). Alkaline phosphatase (ALP) activity was determined using the *p*-nitrophenyl disodium phosphate method.^31^ Mg^2+^ and Cd^2+^ concentrations were analyzed by flame atomic absorption spectrophotometry (AAS; AA-6300C, Shimadzu, Japan). To simplify the calculation, the slight uptake of Mg^2+^ by bacterial cells was neglected, and the decrease in Mg^2+^ concentration in the culture medium was assumed to be entirely attributable to solid-phase precipitation. The mineral yield was calculated according to eqn (6):6
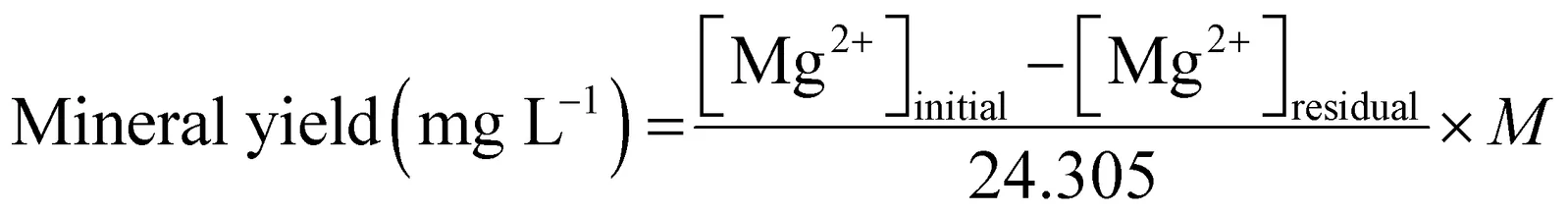
where [Mg^2+^]_initial_ and [Mg^2+^]_residual_ represent the initial and residual Mg^2+^ concentrations in the culture medium (mg L^−1^), respectively, and *M* is the molar mass of the resulting mineral.

The NH_4_^+^ concentration in solution was determined using the phenol–hypochlorite method. PO_4_^3−^ and TP concentrations were measured using the ammonium molybdate spectrophotometric method according to the Chinese national standard GB 11893-89. The TP removal and recovery efficiencies in the culture medium were calculated using eqn (7) and (8), respectively:7
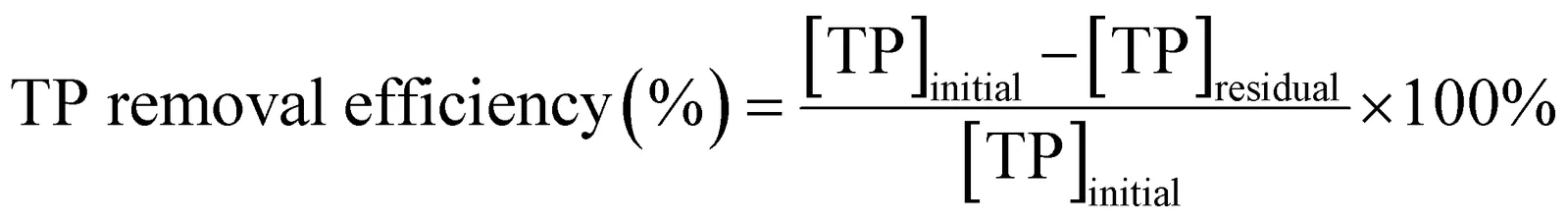
8
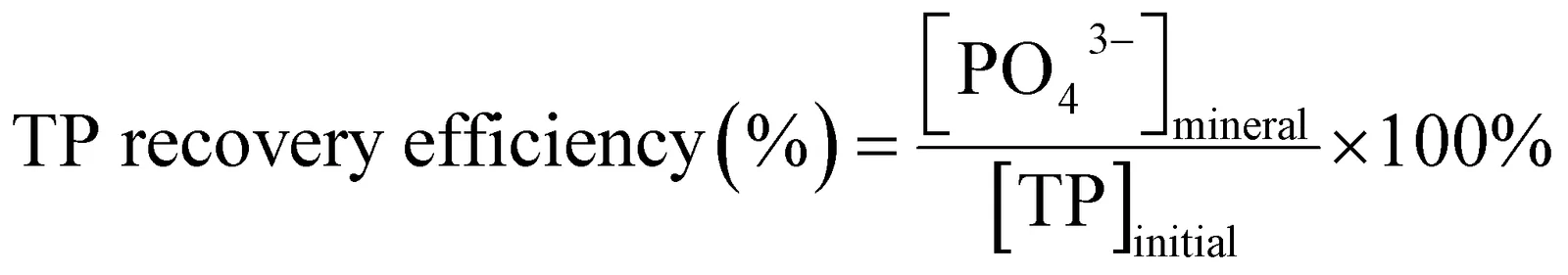
where [TP]_initial_ and [TP]_residual_ are the initial and residual TP concentrations in the culture medium (mmol L^−1^), respectively, and [PO_4_^3−^]_mineral_ is the PO_4_^3−^ content in the mineral (mmol L^−1^).

#### 2.5.2. Solid-phase characterization

The mineral composition of the harvested precipitates was analyzed using an X-ray diffractometer (BTX-526, Olympus, USA), and phase identification was performed using MDI Jade 6.0 software. The micromorphology and elemental composition of the precipitates were characterized by scanning electron microscopy (SEM; Sigma 300, Zeiss, Germany) coupled with energy-dispersive spectroscopy (EDS; Xplore30, Oxford, UK). Fourier-transform infrared spectroscopy (FTIR; Vertex 70, Bruker, Germany) was used to obtain the infrared spectra of the precipitates. Thermal stability was evaluated by thermogravimetric-differential thermal analysis (TG-DTA; STA 7300, Hitachi, Japan) over a temperature range of 25–800 °C at a heating rate of 10 °C min^−1^. X-ray photoelectron spectroscopy (XPS; Nexsa, Thermo Scientific, USA) was used to analyze the surface chemical composition and chemical speciation of the precipitates.

#### 2.5.3. Estimation of struvite content based on NH_4_^+^

According to the stoichiometric formula of struvite, the molar ratio of Mg^2+^, PO_4_^3−^, and NH_4_^+^ in struvite is 1 : 1 : 1. NH_4_^+^ can only precipitate alongside Mg^2+^ and PO_4_^3−^ in the form of struvite, whereas Mg^2+^ and PO_4_^3−^ may also form other compounds (*e.g.*, MgHPO_4_·3H_2_O, Mg_3_(PO_4_)_2_, *etc.*).^32,33^ Therefore, the NH_4_^+^ content of the precipitate can serve as a primary indicator for estimating struvite content.^32^ After the precipitate was dissolved in 0.1 M HCl, the NH_4_^+^ concentration was measured. Struvite content was then calculated using eqn (9):9
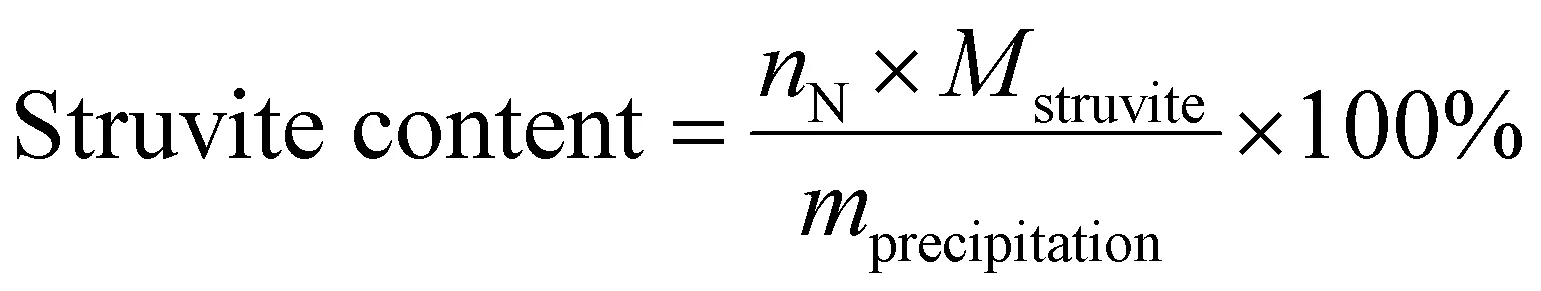
where *n*_N_ is the molar amount of nitrogen (mol), *M*_struvite_ is the molar mass of struvite (g mol^−1^), and *m*_precipitation_ is the mass of the precipitate (g).

#### 2.5.4. TEM observation of MR-1 and extraction of its EPS

Transmission electron microscopy (TEM) observation of MR-1 was performed using bacterial ultrathin sections prepared according to the method reported by Li *et al.*^34^ The microstructure and elemental distribution were examined using high-resolution TEM (FEI-Talos F200S, USA) equipped with EDS.

EPS was extracted from MR-1 using a heating method, as described in Text S1 of the SI. The extracted EPS was analyzed by three-dimensional excitation-emission matrix (EEM) fluorescence spectroscopy (F-7000, HITACHI, Japan) to detect humic acid-like and protein-like substances. The excitation (*E*_x_) and emission (*E*_m_) wavelengths were scanned over ranges of 200–450 nm and 200–550 nm, respectively.

## 3. Results and discussion

### 3.1. Effects of Cd^2+^ on bacterial growth and metabolism in the presence and absence of seawater

First, the effects of different Cd^2+^ concentrations on bacterial growth and metabolism were evaluated in the presence and absence of seawater. The results are shown in Fig. 1. At Cd^2+^ concentrations of 0–10 mg L^−1^, all treatment groups displayed similar growth kinetics. However, at 20–30 mg L^−1^ Cd^2+^, bacterial growth was significantly inhibited, and a pronounced lag phase was observed (Fig. 1a and b). Notably, bacterial tolerance to Cd^2+^ was significantly higher in the biomineralization system (BIS; Fig. 1a) than in the control group (CK, Fig. 1b).

**Fig. 1:**
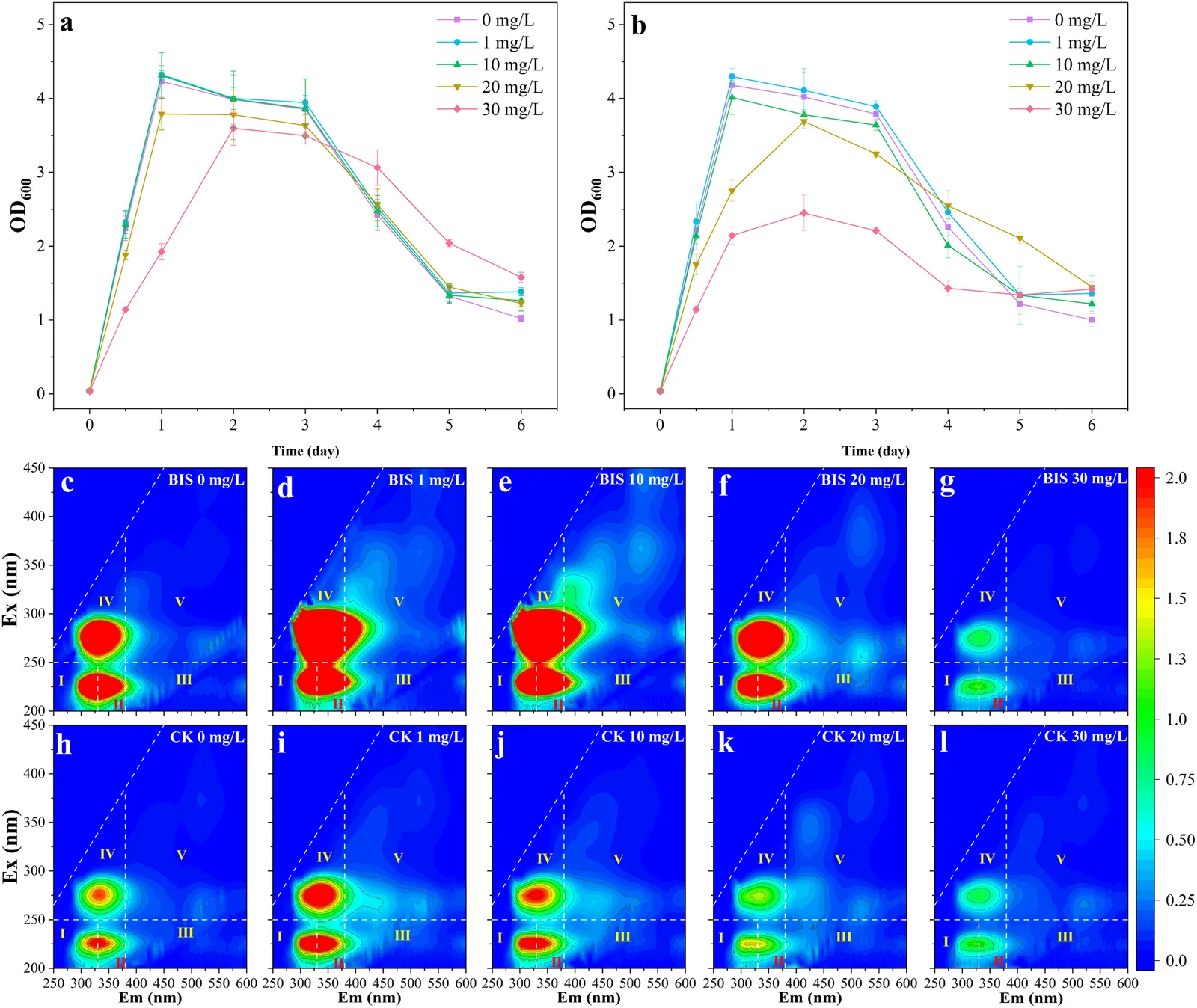
Bacterial growth characteristics and EPS composition in the biomineralization system and control group under Cd^2+^ stress. (a and b) Growth curves of the BIS and CK groups under 0–30 mg L^−1^ Cd^2+^; (c–l) EEM spectra of EPS after 6 days of incubation. Peaks I and II correspond to tyrosine-like and tryptophan-like protein substances, respectively;^37,38^ peaks III and IV represent fulvic acid-like and soluble microbial byproduct-like substances;^38^ and peak V indicates humic acid-like substances.

EEM spectroscopy was further used to characterize the composition and relative abundance of bacterial EPS under different Cd^2+^ concentrations (Fig. 1c–l and S2). The spectral regions identified in the EEM plots are summarized in Table S2. The results showed that the characteristic peak positions were generally consistent across all treatment groups, whereas peak intensities differed markedly. This finding indicates that Cd^2+^ concentration mainly affected the amount of EPS secreted rather than its composition. At all tested Cd^2+^ levels, EPS fluorescence intensity was higher in the BIS group. The higher EPS fluorescence intensity observed in the BIS may reflect an integrated bacterial response to the seawater-containing culture matrix, in which Mg^2+^, other seawater ions, ionic strength, osmotic conditions, and mineralization-associated changes may act simultaneously, making the response difficult to attribute to any single factor.^22,34,35^ The current experimental design does not allow the contribution of each factor to be independently distinguished. Previous studies have shown that bacterial EPS can bind heavy-metal ions outside the cell membrane, thereby reducing their direct contact with cells and alleviating metal toxicity.^36^ Accordingly, the stronger EPS response observed in the BIS may contribute to greater Cd^2+^ tolerance and may partly explain the stronger adaptation to Cd stress relative to the CK group. In addition, Fig. S2 shows that, compared with the Cd-free treatment, exposure to 1–20 mg L^−1^ Cd^2+^ increased the fluorescence intensity of EPS components, indicating that low-to-moderate Cd stress itself may also induce an EPS response.

In addition to the EPS response, temporal monitoring of pH and NH_4_^+^ showed that MR-1 growth was accompanied by NH_4_^+^ accumulation and progressive alkalization of the system (Fig. S1). Higher Cd^2+^ concentrations delayed NH_4_^+^ accumulation and the increase in pH during the early stage of incubation, consistent with the prolonged bacterial lag phase. As MR-1 gradually adapted to Cd stress, both NH_4_^+^ concentration and pH subsequently increased. This pattern is consistent with bacterial metabolism of nitrogen-containing organic compounds and the release of ammonia, which can continuously supply NH_4_^+^ required for struvite formation and establish a mildly alkaline mineralization environment.^22^ As the system became progressively more alkaline, the activity and bioavailability of free Cd^2+^ may have decreased, thereby alleviating inhibition of MR-1 metabolism and favoring continued NH_4_^+^ accumulation and a further increase in pH. This may partly explain why, after 6 days of incubation, the pH and NH_4_^+^ levels under 30 mg L^−1^ Cd^2+^ stress gradually approached those observed in the lower-Cd treatments.

### 3.2. Effects of different Cd^2+^ concentrations on struvite biomineralization

After 6 days of incubation under different Cd^2+^ concentrations, substantial white precipitates accumulated at the bottom of the medium in the BIS group, whereas no visible precipitates formed in the CK group. This result indicates that ions in seawater played a key role in precipitate formation. XRD analysis revealed that the precipitates obtained from the BIS group under different Cd^2+^ concentrations exhibited consistent diffraction peaks corresponding to pure-phase struvite (PDF#71-2089) with the space group *Pmn*2_1_ (Fig. 2a), and no additional Cd-bearing crystalline phase was detected. Further estimation based on the stoichiometric relationship between NH_4_^+^ and struvite yielded struvite contents of approximately 96.03–97.36% across the Cd^2+^ treatments (Table S3), with no significant differences among groups. This indicates that, within the tested concentration range, Cd^2+^ stress did not significantly affect the relative struvite content of the precipitates induced by MR-1. FTIR analysis further confirmed that the precipitates were struvite (Fig. 2b), as their characteristic peaks were consistent with those reported previously.^15,39,40^ Notably, no disappearance, positional shift, splitting, or newly formed absorption peaks were observed in the FTIR spectra, indicating that Cd^2+^ was not substantially incorporated into the struvite crystal lattice. Furthermore, thermogravimetric analysis showed that all samples exhibited similar thermal stability, with weight losses close to the theoretical value of 51.42% for struvite (Fig. S3).^15^

**Fig. 2:**
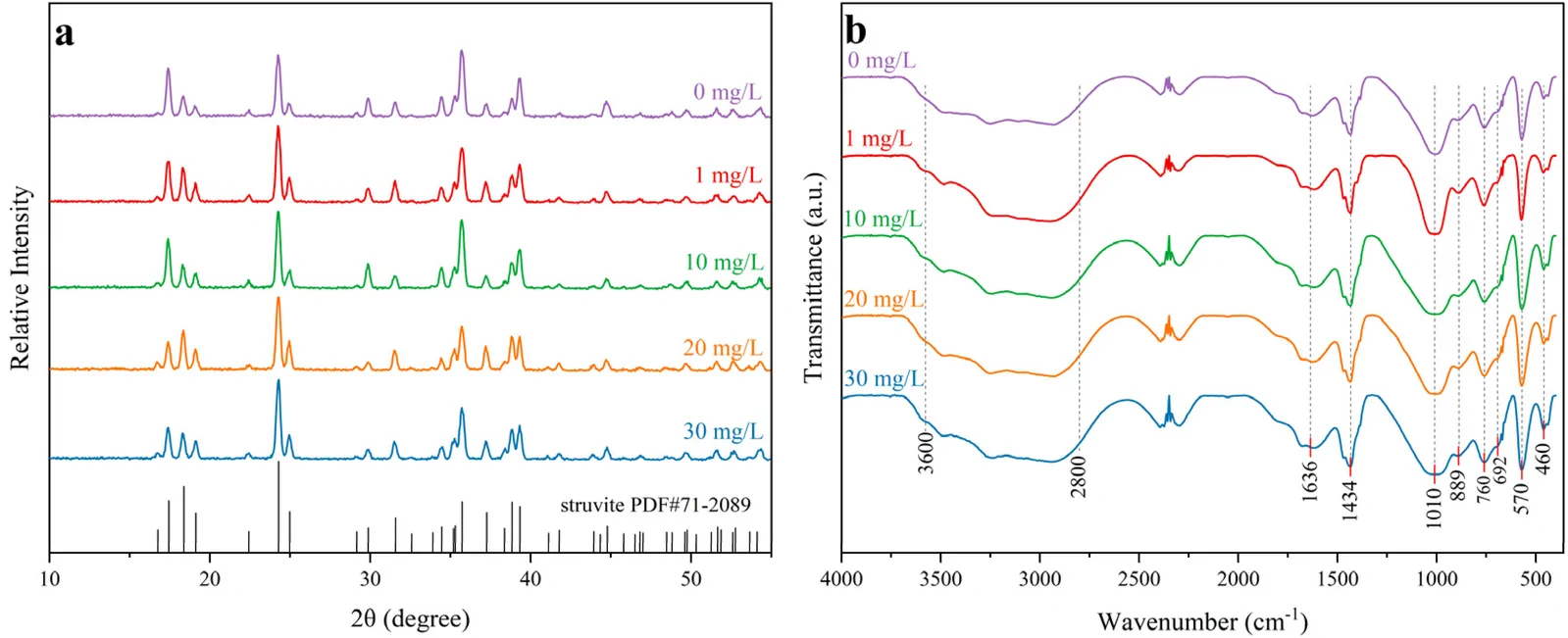
XRD patterns (a) and FTIR spectra (b) of precipitates from bacterial mineralization under 0–30 mg L^−1^ Cd^2+^.

High Cd^2+^ concentrations inhibited alkaline phosphatase activity, which is associated with organic phosphorus mineralization (Fig. S4a), thereby reducing TP removal efficiency as Cd^2+^ levels increased (Fig. S4b). Notably, even under extreme high-Cd^2+^ stress, the BIS retained higher ALP activity than the CK group, which may be related to the stronger EPS response observed under the seawater-containing conditions. The relatively abundant EPS may immobilize Cd^2+^ extracellularly and reduce its direct toxicity to cells, thereby helping to maintain bacterial metabolic activity and ALP function. Although phosphate dynamics were not continuously monitored in this study, a previous study showed that bacteria preferentially utilized available inorganic phosphorus, while depletion of inorganic phosphorus stimulated ALP activity and promoted the hydrolysis of organic phosphorus, thereby replenishing inorganic phosphate available for struvite formation.^22^ This process may partly explain why the BIS maintained a TP removal efficiency of 88.27–91.41% under Cd^2+^ stress. Of the initial TP, 67.35–70.64% was ultimately recovered as struvite (Table S4), indicating that a portion of the removed phosphorus did not enter the recoverable struvite fraction. This phosphorus may have been assimilated by MR-1 cells for growth and metabolism or immobilized through adsorption to EPS or cell-surface functional groups. In addition, although XRD did not detect obvious additional crystalline phosphorus-containing minerals, the presence of small amounts of low-abundance or amorphous phosphorus-containing solids cannot be completely excluded. In contrast, the TP removal efficiency of the CK group was only approximately 19%, consistent with the lack of sufficient Mg^2+^ for struvite precipitation. Under the experimental conditions, the chemical precipitation system exhibited a TP removal efficiency of 58.12–60.23%, which may partly reflect the absence of microbially mediated conversion of organic phosphorus into mineralizable inorganic forms.

SEM analysis was further performed to investigate the effects of Cd^2+^ on the morphology and structure of struvite crystals. As shown in Fig. 3a–e, the morphology of bacterially mineralized struvite (bio-struvite) remained largely unchanged across different Cd^2+^ concentrations, consistently displaying typical roof-like, coffin-lid-like, trapezoidal, plate-like, and prismatic shapes. This complex morphological assemblage differed from those previously reported for MR-1-induced mineralization,^32,41^ suggesting that the complex ionic composition of seawater may influence bacterial growth and metabolism and, in turn, affect crystal growth. Overall, the bio-struvite crystals retained relatively well-defined structures and smooth surfaces, and no Cd signal was detected by EDS in the bio-struvite samples (Fig. 3f and S5), indicating no obvious Cd enrichment on the analyzed crystal surfaces, although trace amounts below the detection limit cannot be excluded. In contrast, chemically precipitated struvite (chem-struvite) displayed smooth crystal faces in the absence of Cd^2+^ (Fig. S6a). However, in the presence of Cd^2+^, the crystal surfaces became rough and showed evident particle accumulation (Fig. S6b). Because the XRD results confirmed that struvite was the only crystalline phase in the chemical precipitation system (Fig. S7), these particles were likely amorphous Cd-containing compounds formed through binding between Cd^2+^ and –OH/–PO_4_ groups.^14^ Numerous studies have demonstrated that heavy metals can influence struvite chemical precipitation through various mechanisms.^12^ For example, Cd^2+^, Cu^2+^, Pb^2+^, and Zn^2+^ have been reported to reduce product purity and modify crystal morphology.^14,42^ Cu^2+^ and Zn^2+^ can also coprecipitate with struvite as Cu–OH, Cu–PO_4_, Cu–NH_3_, Zn–PO_4_, or Zn–OH species,^43,44^ whereas Ni^2+^ can substitute for Mg^2+^ to form Ni-struvite (NiNH_4_PO_4_·6H_2_O).^45^ Overall, these experimental observations indicate that, relative to the chemical precipitation system, bacterial biomineralization limited the transfer of Cd into the recovered struvite.

**Fig. 3:**
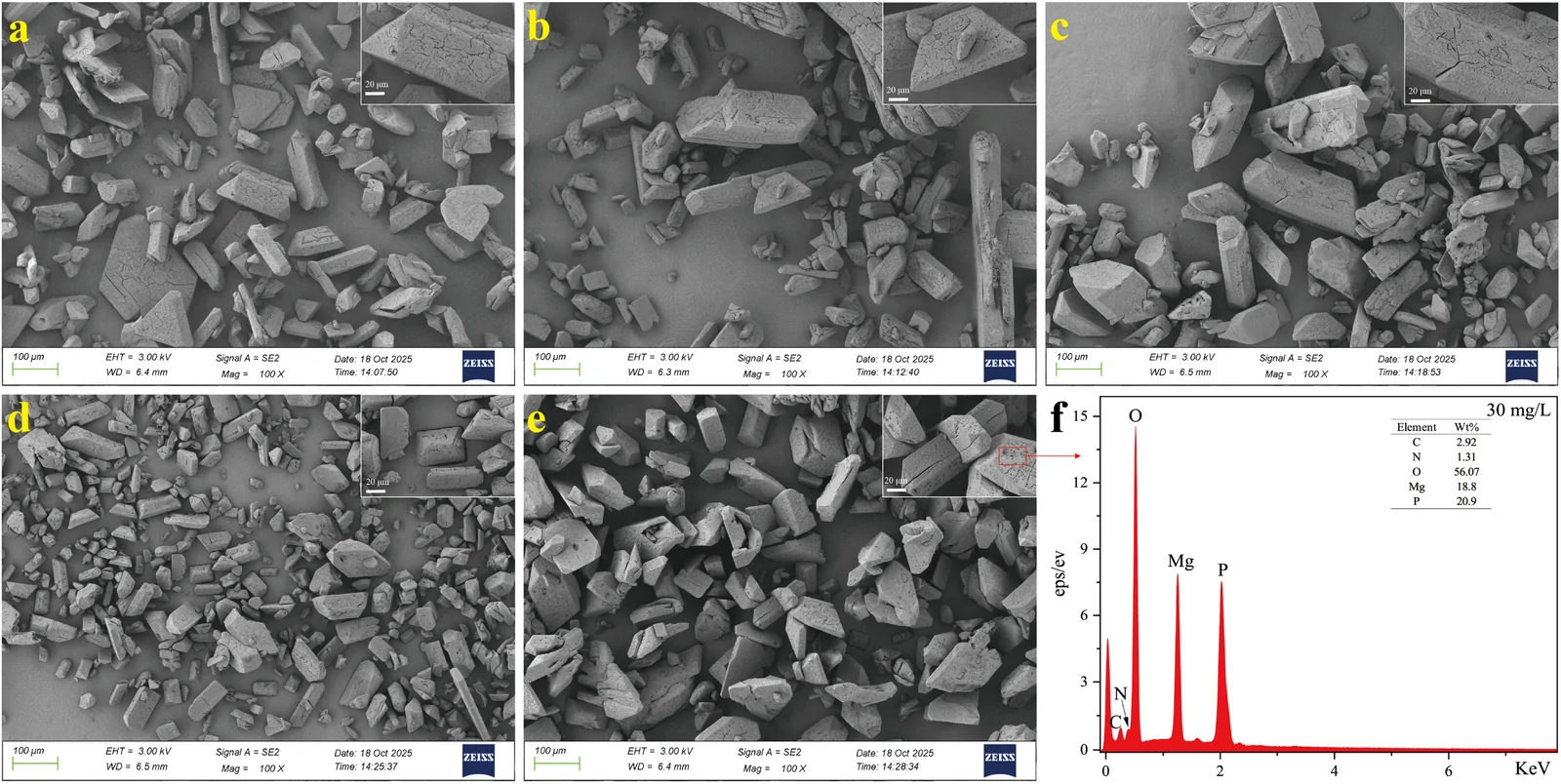
Morphology and elemental composition of bio-struvite under different Cd^2+^ concentrations. (a–e) SEM images of struvite obtained *via* bacterial mineralization at 0–30 mg L^−1^ Cd^2+^; (f) EDS spectrum of a crystal obtained at 30 mg L^−1^ Cd^2+^. Insets show high-magnification images of the corresponding crystals.

Overall, XRD, FTIR, TG-DTA, SEM-EDS, and stoichiometric calculations collectively showed that MR-1 maintained a struvite-dominated mineralization process across the entire Cd^2+^ stress gradient. Meanwhile, the decreases in ALP activity and TP removal efficiency at the highest Cd^2+^ level indicate that extreme Cd stress still exerted some inhibition on microbially mediated phosphorus transformation. Thus, the BIS maintained relatively stable struvite formation under Cd^2+^ stress, although this process was jointly regulated by microbial metabolism and the complex ionic environment of seawater. Future studies using pure Mg-salt controls with equivalent Mg^2+^ concentrations and artificial seawater could further distinguish the specific contributions of individual seawater components and ionic conditions to microbial mineralization.

### 3.3. Content and speciation of Cd^2+^ in struvite and bacterial cells

Although Cd was not detected on the surface of bio-struvite (Fig. S5), this was likely because its content was below the detection limit of the instrument. To quantify Cd incorporation, the struvite samples were dissolved in 1 mol L^−1^ HCl, and their Cd content was measured using inductively coupled plasma mass spectrometry (ICP-MS). As shown in Table 1, at different initial Cd^2+^ concentrations (1, 10, 20, and 30 mg L^−1^), the Cd content in both bio-struvite and chem-struvite increased as the initial Cd^2+^ concentration increased. Under their respective experimental conditions, the measured Cd content of bio-struvite was significantly lower than that of chem-struvite at each tested initial Cd^2+^ concentration. At an initial Cd^2+^ concentration of 30 mg L^−1^, the Cd content in bio-struvite was only 354.35 ± 3.80 mg kg^−1^, whereas that in chem-struvite reached 18 339.83 ± 793.88 mg kg^−1^, approximately 51.8-fold higher. These results indicate that, under the present experimental conditions, the presence of MR-1 effectively limited Cd transfer into the recovered struvite, although product safety remained strongly dependent on the initial Cd^2+^ concentration. When the initial Cd^2+^ concentration was 1 mg L^−1^, the Cd content of bio-struvite was 7.13 ± 1.38 mg kg^−1^, below the 10 mg kg^−1^ limit specified in GB 38400-2019; however, when the initial Cd^2+^ concentration was ≥10 mg L^−1^, the Cd content of the product exceeded this limit. Therefore, the high-Cd stress experiments were primarily intended to evaluate the capacity and mechanistic limits of the biomineralization system in restricting Cd transfer to the mineral phase, rather than to imply the agricultural safety of products obtained under high-Cd conditions. For wastewater with elevated Cd^2+^ concentrations, the aqueous Cd^2+^ level should be reduced before struvite recovery, or the recovered mineral should be further purified and subjected to safety assessment before resource utilization is considered.

**Table 1:** Content of Cd^2+^ in bio-struvite and chem-struvite^a^

		Cd^2+^ initial concentration (mg L^−1^)			
		1	10	20	30
Content of Cd^2+^ in struvite (mg kg^−1^)	Bio-struvite	7.13 ± 1.38	97.93 ± 3.50	347.50 ± 16.98	354.35 ± 3.80
	Chem-struvite	923.74 ± 3.24*	5257.08 ± 138.93*	8363.96 ± 197.43*	18 339.83 ± 793.88*

a Data are presented as the mean ± standard deviation (*n* = 3). The asterisk (*) indicates a significant difference in Cd content between bio-struvite and chem-struvite at the same initial Cd^2+^ concentration, as determined by an independent-samples *t*-test (*p* < 0.05).

Further analysis of aqueous Cd^2+^ showed that, at initial Cd^2+^ concentrations of 1–10 mg L^−1^, both the BIS and CK groups achieved nearly complete Cd^2+^ removal. As the Cd^2+^ concentration increased, removal efficiency gradually declined, consistent with the stronger inhibition of bacterial growth at higher Cd^2+^ levels (Fig. 4a). Nevertheless, the BIS maintained higher Cd^2+^ removal efficiency than the CK group across the entire tested concentration range. Together with the EEM results, the stronger EPS response in the BIS may be associated with its greater Cd^2+^ tolerance and removal capacity. Phase-distribution analysis further showed that only 0.58–1.40% of Cd entered the mineral phase with struvite, whereas 84.24–99.42% of the removed Cd was associated with the bacterial fraction (Fig. 4b), indicating that MR-1 cells and their surface components were the major carriers of Cd immobilization in the BIS and helped limit Cd transfer into the recovered struvite.

**Fig. 4:**
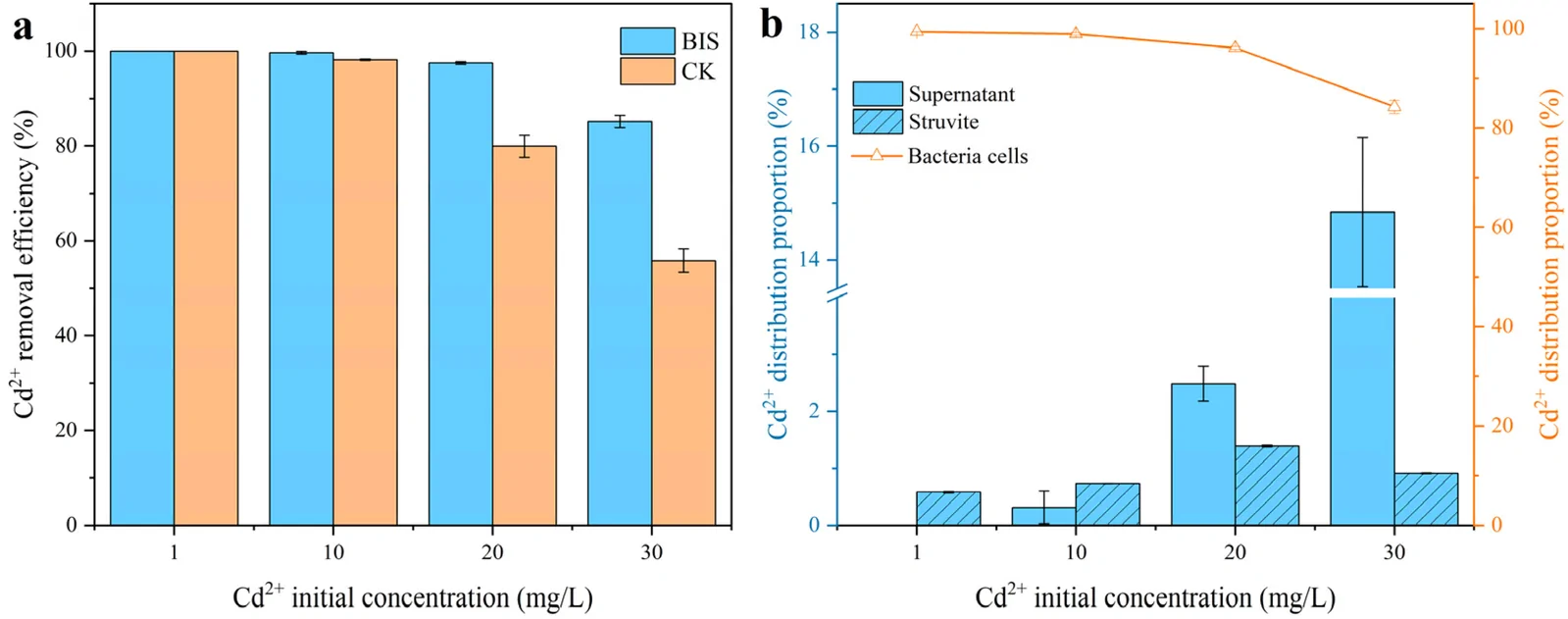
Cd^2+^ removal efficiency and distribution among different phases of the biomineralization system. (a) Cd^2+^ removal efficiency as a function of the initial Cd^2+^ concentration in the BIS and CK groups; (b) distribution of Cd^2+^ among the supernatant, struvite, and bacterial cells within the BIS. The Cd^2+^ content associated with bacterial cells was calculated by subtracting the Cd retained in struvite and the residual Cd^2+^ in the supernatant from the total amount of Cd^2+^ initially added.

XPS was further used to analyze the chemical state of Cd on the surfaces of different samples (Fig. 5). At an initial Cd^2+^ concentration of 30 mg L^−1^, no obvious Cd 3d characteristic peaks were detected on the surface of bio-struvite (Fig. 5a, b, and S8). In contrast, distinct Cd 3d_5/2_ (405.73 eV) and Cd 3d_3/2_ (412.46 eV) doublet signals were observed in chem-struvite (Fig. 5c and d). These binding energies are consistent with those reported for cadmium phosphate.^46^ In bacterial samples isolated from the BIS (Fig. 5e and f), characteristic Cd3d peaks at 404.74 eV and 411.36 eV were also detected. These results indicate that Cd in the BIS was predominantly bound to bacterial components. Taken together with the other characterization results, XRD and FTIR showed no obvious changes in crystal structure or characteristic bands attributable to Cd incorporation, suggesting that substantial Cd incorporation into the bio-struvite lattice did not occur, although trace lattice substitution cannot be excluded. Neither SEM-EDS nor XPS showed obvious Cd enrichment on the external surface of bio-struvite, whereas ICP-MS still detected a small amount of Cd after complete acid dissolution of the bulk sample. Collectively, this Cd may be associated with pore entrapment within crystal aggregates or encapsulation of Cd-bearing bacterial cells or EPS during nucleation and growth, and may also include a minor contribution from lattice incorporation. However, the available evidence does not allow these occurrence forms to be quantitatively distinguished. Overall, Cd was preferentially retained in the bacterial fraction rather than transferred to the recovered struvite phase.

**Fig. 5:**
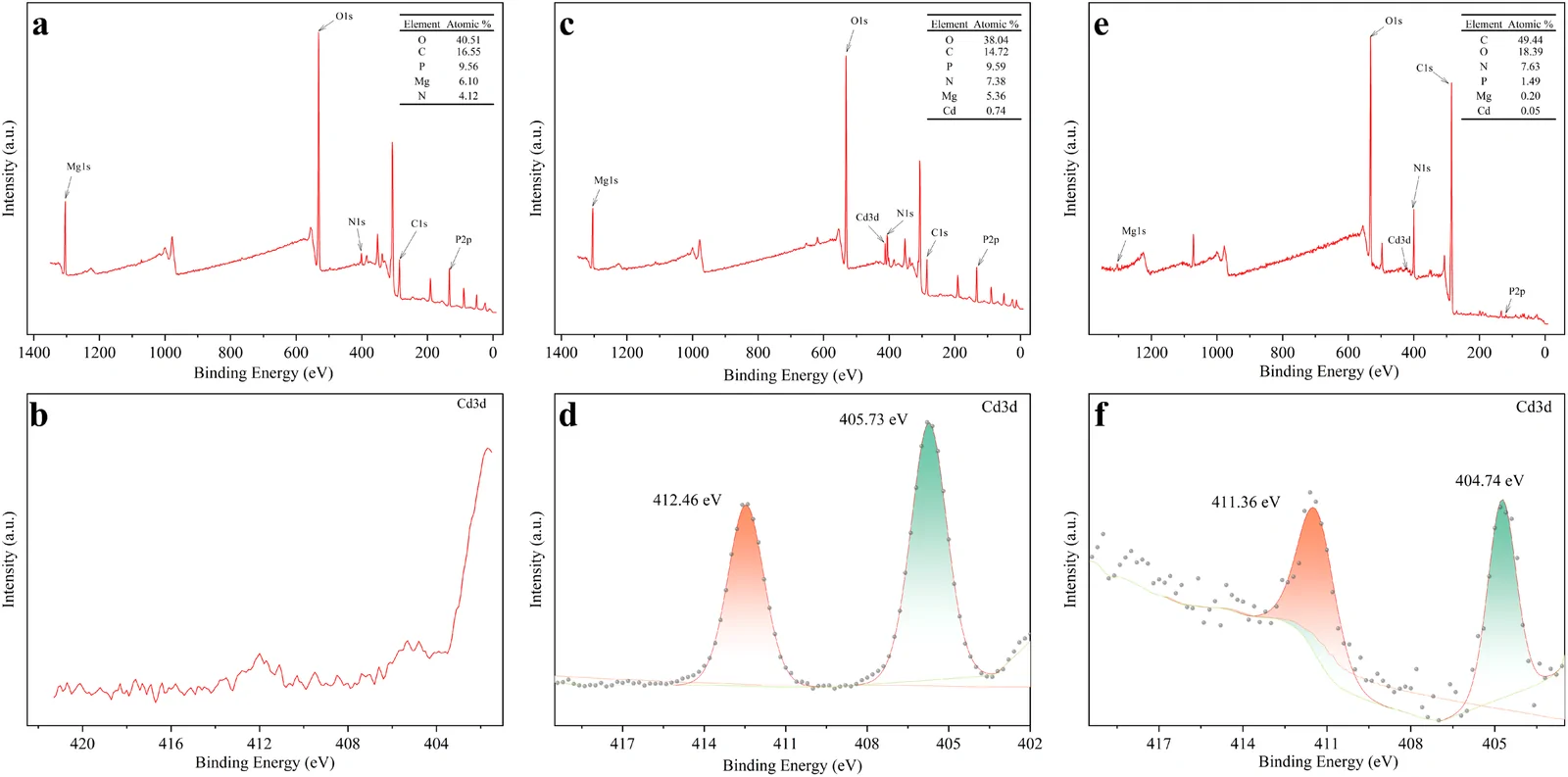
XPS survey and high-resolution Cd 3d spectra of samples obtained under a Cd^2+^ concentration of 30 mg L^−1^. (a and b) Bio-struvite; (c and d) chem-struvite; (e and f) bacterial cells isolated from the biomineralization system.

### 3.4. Mechanism of Cd^2+^ biosorption by MR-1

#### 3.4.1. Analysis of adsorption isotherm models

To investigate the biosorption potential of MR-1 for Cd^2+^ in the biomineralization system, biosorption experiments were performed using MR-1 biomass as the biosorbent. As shown in Fig. 6a, the adsorption capacity of MR-1 increased significantly with increasing initial Cd^2+^ concentration, whereas removal efficiency decreased. This pattern was mainly attributable to the relatively abundant active sites at low Cd^2+^ concentrations, which resulted in higher removal efficiency but lower adsorption capacity. Conversely, at elevated Cd^2+^ concentrations, the adsorption sites gradually became saturated, increasing the adsorption capacity per unit mass but reducing the removal efficiency.^24,29^

**Fig. 6:**
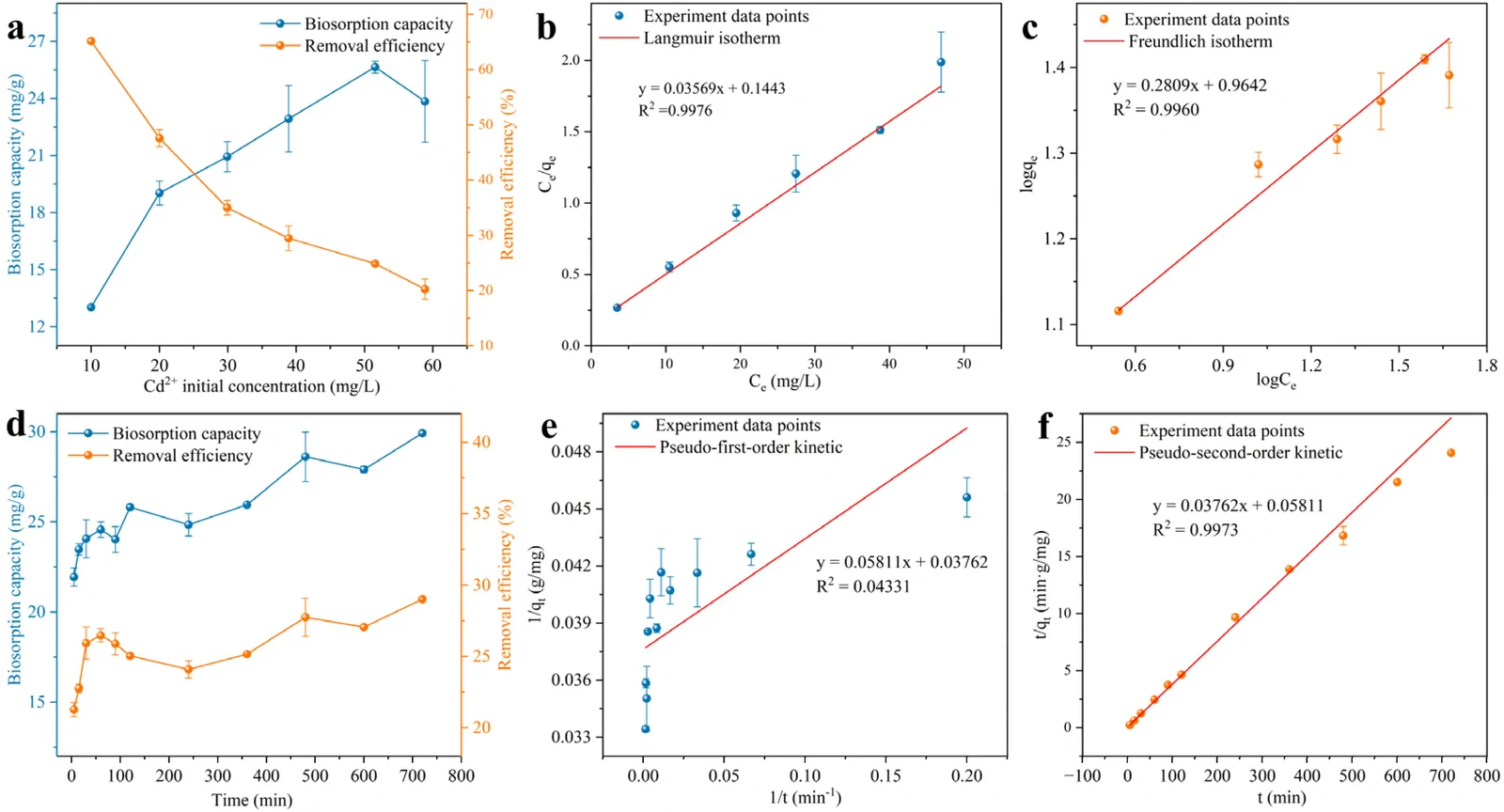
Cd^2+^ adsorption performance of MR-1 and fitting with isotherm and kinetic models. (a) Effect of initial Cd^2+^ concentration on the adsorption capacity and Cd^2+^ removal efficiency of MR-1; (b and c) Langmuir and Freundlich adsorption isotherm fitting plots; (d) effect of contact time on the adsorption capacity and Cd^2+^ removal efficiency of MR-1; (e and f) pseudo-first-order and pseudo-second-order kinetic fitting plots.

The Cd^2+^ adsorption behavior and adsorption capacity of MR-1 were further characterized using two commonly applied isotherm models: the Langmuir model (eqn (2)) and the Freundlich model (eqn (3)). As shown in Fig. 6b, c and Table S5, both models adequately described Cd^2+^ adsorption onto MR-1, with high goodness of fit (*R*^2^ = 0.9976 for the Langmuir model and *R*^2^ = 0.9960 for the Freundlich model). Although the goodness-of-fit values were similar, the Langmuir model provided a slightly better fit, indicating that Cd^2+^ adsorption mainly followed a monolayer process with a certain degree of surface heterogeneity.^24^ In addition, the maximum biosorption capacity (*q*_max_) predicted by the Langmuir model was 28.02 mg g^−1^, which is comparable to the Cd^2+^ adsorption capacities reported for other biosorbents (Table S6). Collectively, MR-1 exhibited strong Cd^2+^ binding ability and favorable surface adsorption properties.

#### 3.4.2. Analysis of adsorption kinetic models

The effect of contact time (5–720 min) on the adsorption behavior of the MR-1 biosorbent was also examined. As shown in Fig. 6d, Cd^2+^ removal followed a typical biphasic kinetic pattern: the adsorption rate was initially rapid but gradually decreased thereafter. The rapid phase was mainly attributed to the abundance of vacant adsorption sites on the MR-1 cell surface; as these sites became saturated, the adsorption rate declined.^23,24,47^ The rapid phase was mainly governed by an energy-independent surface adsorption mechanism, which dominated throughout the Cd^2+^ removal process, followed by a slower intracellular accumulation phase.^48^

To further elucidate the interaction characteristics between MR-1 and Cd^2+^, adsorption kinetics were modeled (Fig. 6e, f and Table S7). The pseudo-second-order kinetic model provided the best fit to the experimental data (*R*^2^ = 0.9973), suggesting a substantial contribution of chemisorption to the overall adsorption process.^23,24^

#### 3.4.3. TEM and FTIR analyses after bacterial Cd^2+^ adsorption

To further elucidate the spatial distribution of Cd^2+^ in bacterial cells, TEM-EDS was performed on ultrathin sections of bacteria exposed to 30 mg L^−1^ Cd^2+^. The dark-field TEM image (Fig. 7a) showed a distinct electron-dense deposition layer on the cell surface, suggesting marked enrichment of the relevant ions in the outer cell layer. Elemental mapping results (Fig. 7b) further revealed that Cd was distributed both on the cell surface and inside the cells. EDS point analysis further showed that the extracellular Cd content was markedly higher than the intracellular level (Fig. S9), confirming that Cd accumulation occurred primarily on the cell surface. In summary, bacterial Cd^2+^ removal occurred predominantly through surface adsorption.

**Fig. 7:**
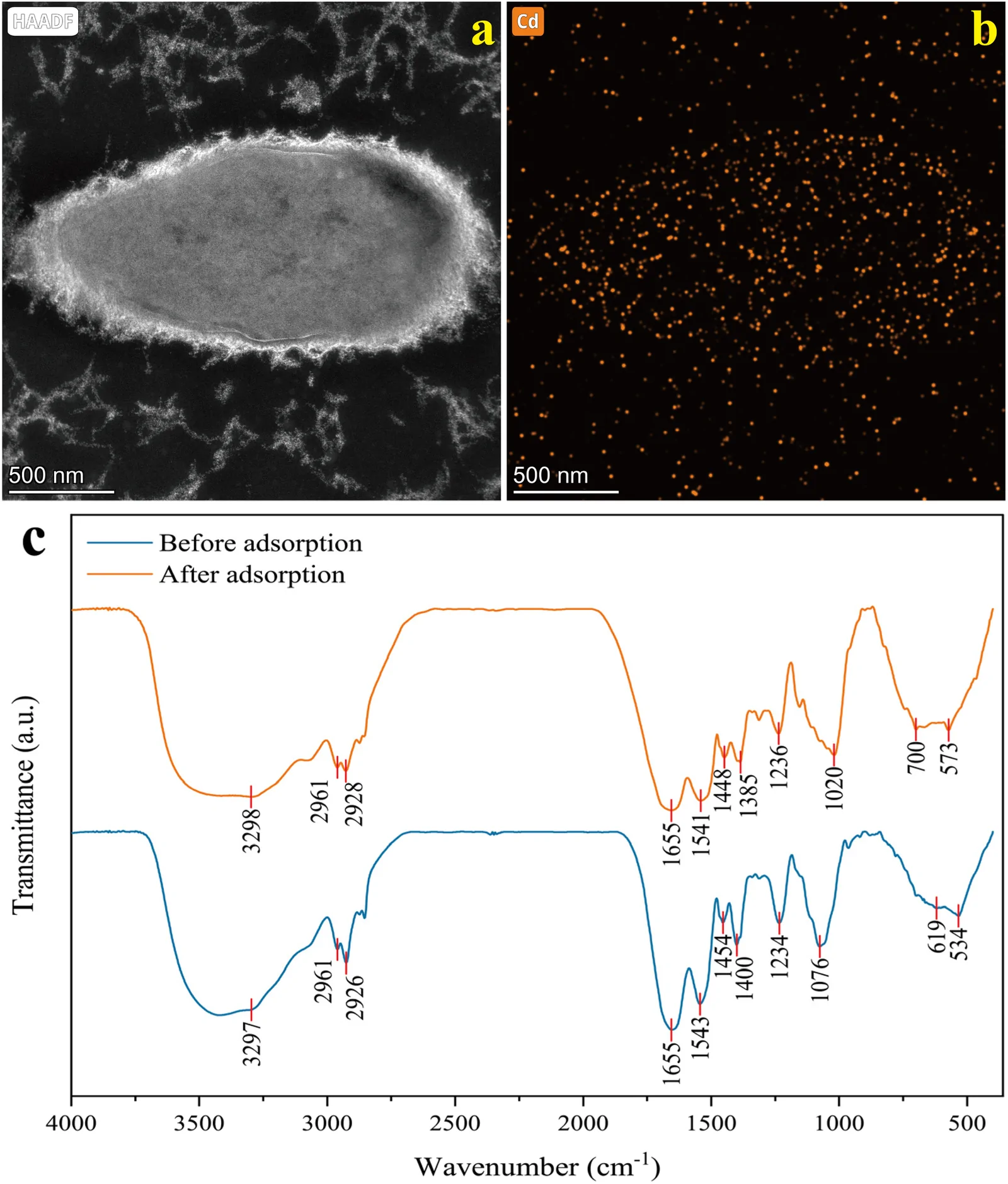
TEM-EDS (a and b) and FTIR (c) analyses of MR-1 biosorbent after adsorption at a Cd^2+^ concentration of 30 mg L^−1^.

FTIR spectroscopy was used to analyze changes in surface functional groups of MR-1 before and after Cd^2+^ adsorption (Fig. 7 and Table S8). The results showed that, after adsorption, the broad absorption band in the range of 3297–3500 cm^−1^ exhibited slight shifts in both position and shape. This region is associated with the stretching vibrations of –OH and –NH groups, implying that these nucleophilic groups interacted with Cd^2+^ during adsorption,^49^ possibly through changes in hydrogen-bonding environments or coordination binding. Furthermore, the amide II vibration peak at 1543 cm^−1^ and the carboxyl-related peak at 1454 cm^−1^ shifted to 1541 and 1448 cm^−1^, respectively, after adsorption. These shifts suggest that amino groups in protein structures and carboxylate groups in the cell wall served as major binding sites for Cd^2+^, further highlighting the crucial role of cell-wall functional groups in metal binding.^25,49^ Notably, the vibration peak associated with polysaccharides or phosphate esters shifted markedly from 1076 cm^−1^ before adsorption to 1020 cm^−1^ after adsorption, accompanied by a change in peak shape. This shift indicates that phosphate ester groups associated with structures such as P–O–C and P=O on the cell surface or within EPS participated in strong coordination or structural rearrangement with Cd^2+^.^50,51^ In addition, the emergence or enhancement of peaks in the low-wavenumber region (700–573 cm^−1^) can be ascribed to metal–ligand vibrations of Cd–O or Cd–P bonds,^25,52^ indicating that Cd^2+^ may form stable coordination complexes with surface phosphate groups or even generate cadmium phosphate-like microprecipitates.

Taken together, the microscopic and spectroscopic results indicate that Cd^2+^ immobilization by MR-1 mainly involved coordination and complexation with hydroxyl, amino, carboxyl, amide, and phosphorus-containing functional groups on the cell surface, and may also have involved ion exchange and Cd–P interactions. The good fit of the pseudo-second-order kinetic model was consistent with these observations and further supported an important role of chemisorption in Cd^2+^ immobilization by MR-1. Overall, immobilization of Cd^2+^ by MR-1 helped reduce Cd transfer into struvite. Therefore, timely and effective separation of cellular components from the mineral product after mineralization is important for simultaneously obtaining struvite with a relatively low Cd content and removing Cd^2+^ from the aqueous phase.

Although this study demonstrated the feasibility of MR-1-mediated biomineralization for simultaneous phosphorus recovery and Cd removal from Cd^2+^-containing, phosphorus-rich wastewater, translation from the laboratory system to practical wastewater treatment requires further validation. On the one hand, the complex ionic environment introduced by natural seawater is strongly coupled with microbial metabolism and mineral nucleation, and their relative contributions to Cd immobilization and phosphorus mineralization remain incompletely resolved. On the other hand, the fate of a fraction of the phosphorus and the occurrence forms of small amounts of Cd in bio-struvite still require further clarification. Future studies should evaluate long-term stability, mineral recovery efficiency, and biomass-mineral separation under complex water-quality conditions in continuous-flow and pilot-scale systems. High-temporal-resolution ion monitoring, multi-omics, and interfacial characterization should also be integrated to elucidate the coordinated regulation of Cd migration and struvite nucleation by microbial metabolism, EPS, and mineral interfaces. In addition, the environmental safety of recovered struvite should be systematically evaluated in terms of heavy-metal stability, phytoavailability, and ecotoxicity, and the approach should be extended to systems containing multiple coexisting metals, with the aim of developing biomineralization strategies that integrate nutrient recovery, selective control of heavy metals, and safe product utilization.

## 4. Conclusion

This study demonstrates that, under an experimental Cd^2+^ stress gradient of 1–30 mg L^−1^, MR-1 can use Mg^2+^ from seawater to induce the formation of struvite-dominated precipitates while simultaneously removing aqueous Cd^2+^. Under the respective experimental conditions, the Cd content of bio-struvite was 7.13–354.35 mg kg^−1^, significantly lower than the 923.74–18 339.83 mg kg^−1^ measured in chem-struvite. TP removal efficiencies in the biomineralization and chemical precipitation systems were 88.27–91.46% and 58.12–60.23%, respectively. Cd phase-distribution analysis showed that most of the removed Cd was associated with the bacterial fraction, with only a small proportion (0.58–1.40%) entering the mineral phase with struvite. TEM-EDS and FTIR analyses indicated that carboxyl, hydroxyl, amino, amide, and phosphorus-containing functional groups on the MR-1 surface participated in Cd^2+^ immobilization through coordination, complexation, and ion exchange, while the good fit of the pseudo-second-order kinetic model further supported an important role of chemisorption in this process. At an initial Cd^2+^ concentration of 1 mg L^−1^, the Cd content of bio-struvite was 7.13 ± 1.38 mg kg^−1^, below the fertilizer limit of 10 mg kg; however, at initial Cd^2+^ concentrations of ≥10 mg L^−1^, the product Cd content exceeded this limit. Therefore, Cd pretreatment, product purification, and safety assessment are still required before resource utilization under high-Cd conditions. Overall, MR-1-mediated biomineralization shows potential for coupling phosphorus recovery with Cd^2+^ removal and provides a new technical approach for the resource-oriented treatment of Cd-contaminated, phosphorus-rich wastewater. Future work should further validate process stability, mineral-separation efficiency, and product environmental safety under complex wastewater and continuous-operation conditions.

## Author contributions

Lei Meng: data curation, investigation, software, writing – original draft, writing – review & editing; Bin Lian: conceptualization, funding acquisition, project administration, resources, supervision, writing – review & editing.

## Conflicts of interest

There are no conflicts to declare.

## Supplementary Material

RA-016-D6RA05024G-s001

## Data Availability

Data will be made available upon request. Supplementary information (SI): Text S1, Fig. S1–S9, and Tables S1–S8. See DOI: https://doi.org/10.1039/d6ra05024g.
