# Supplementary material for: Bacterially induced synthesis of low-Cd struvite coupled with Cd2+ biosorption under high cadmium stress
Source: RSC Adv. 2026 Sep 21;16(44):55952–63. doi: 10.1039/d6ra05024g (PMC13591419; doi:10.1039/d6ra05024g)
Supplement: Supplementary file 1 [file RA-016-D6RA05024G-s001.pdf]

Supplementary Information (SI) for RSC Advances.
This journal is © The Royal Society of Chemistry 2026

Supporting data

Bacterially induced synthesis of low-Cd struvite coupled with Cd^{2+} biosorption under high cadmium stress

Lei Meng[a] and Bin Lian*[b]

[a] College of Life Science, Nanjing Normal University, Nanjing 210023, China

[b] Jiangsu Key Laboratory of Ocean-Land Environmental Change and Ecological Construction, College of Marine Science and Engineering, Nanjing Normal University, Nanjing 210023, China

*Corresponding author:

Bin Lian, E-mail: bin2368@vip.163.com

Text

Text S1. Extraction method of bacterial EPS

(1) After 6 days of cultivation, 40 mL of bacterial fermentation broth from each treatment group was transferred into centrifuge tubes and centrifuged at 1200 × *g* for 10 min. (2) The supernatant was discarded, and the bacterial cells were resuspended in 20 mL of sterile distilled water, followed by centrifugation at 1200 × *g* for 10 min. This washing step was repeated twice to remove residual culture medium from the cells. (3) Subsequently, the bacterial cells were resuspended in 40 mL of sterile water and heat-treated in a water bath at 60 °C for 30 min, followed by centrifugation at 13800 × *g* for 10 min. (4) Finally, the supernatant was filtered through a 0.22 μm cellulose acetate membrane to obtain a colorless, transparent EPS solution. (5) The resulting EPS solution was used for three-dimensional excitation-emission matrix fluorescence spectroscopy analysis.

Figures

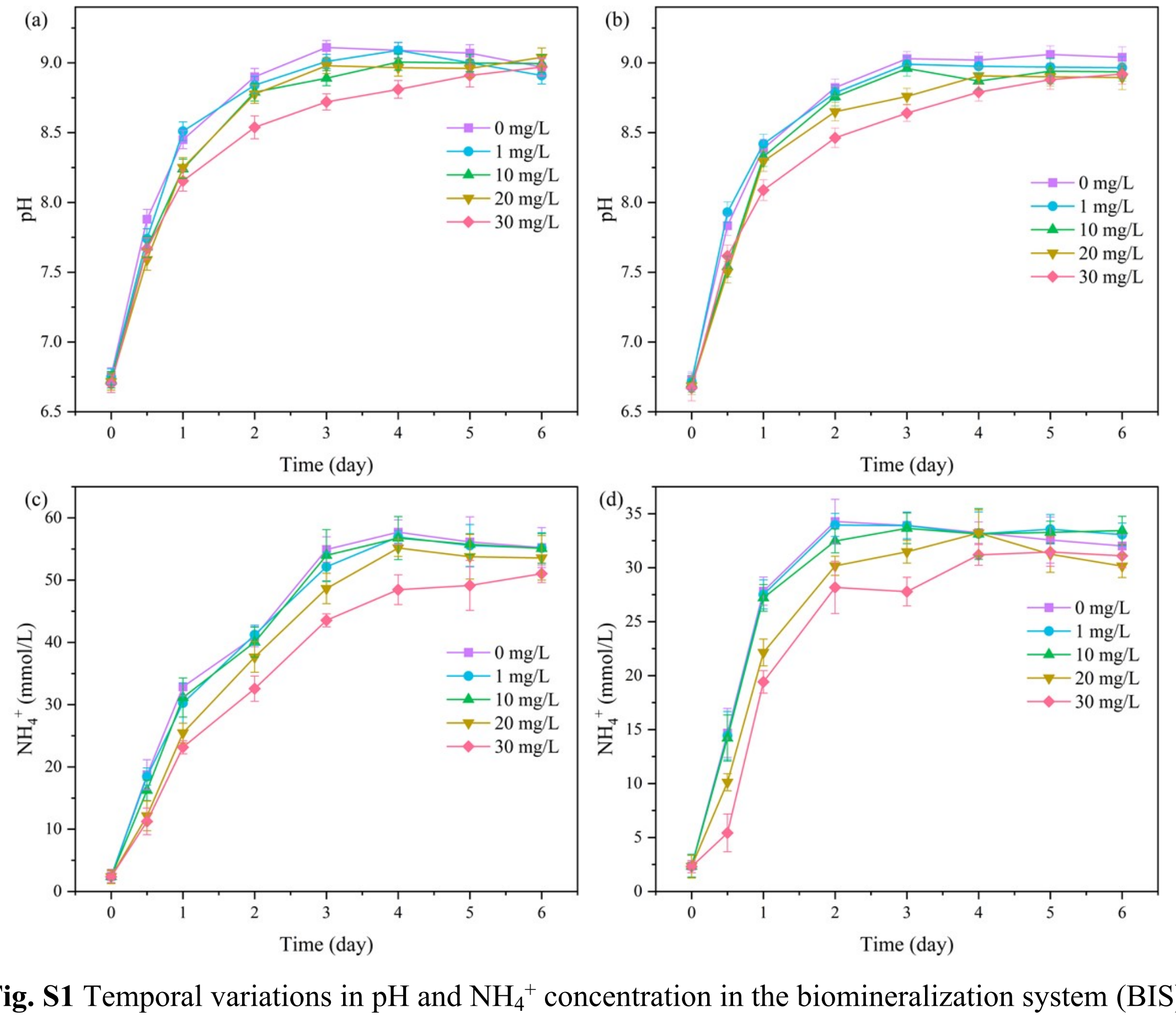

Fig. S1 Temporal variations in pH and NH_4^+ concentration in the biomineralization system (BIS) and control group (CK) under different Cd^{2+} concentrations. (a) pH in the BIS; (b) pH in the CK; (c) NH_4^+ concentration in the BIS; and (d) NH_4^+ concentration in the CK during 6 days of incubation.

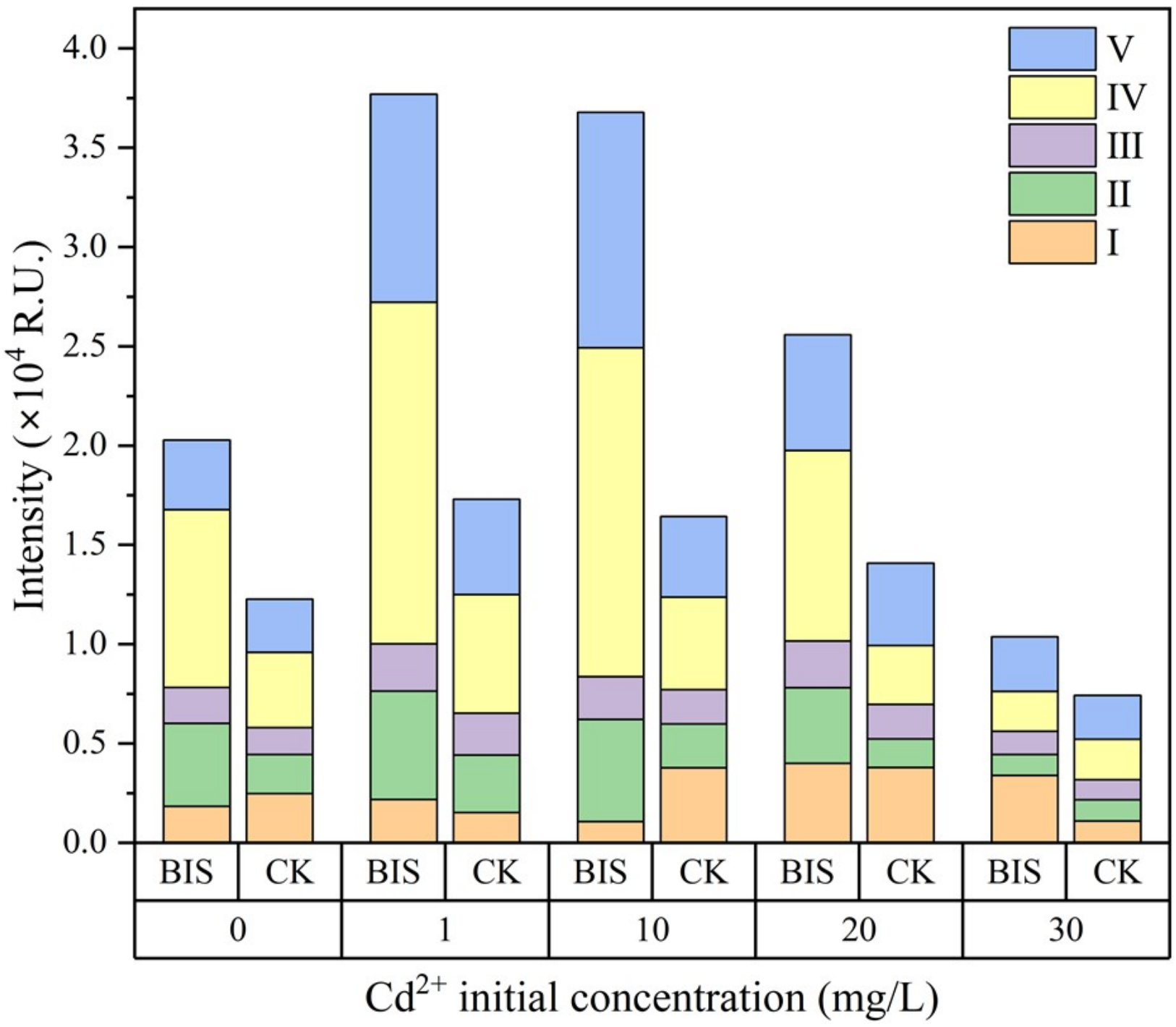

Fig. S2 Fluorescence intensities of the five fluorescent components in the BIS and CK groups at Cd^{2+} concentrations of 0-30 mg/L. I: Tyrosine-like protein substances; II: Tryptophan-like protein substances; III: Fulvic acid-like substances; IV: Soluble microbial byproduct-like substances; V: Humic acid-like substances.

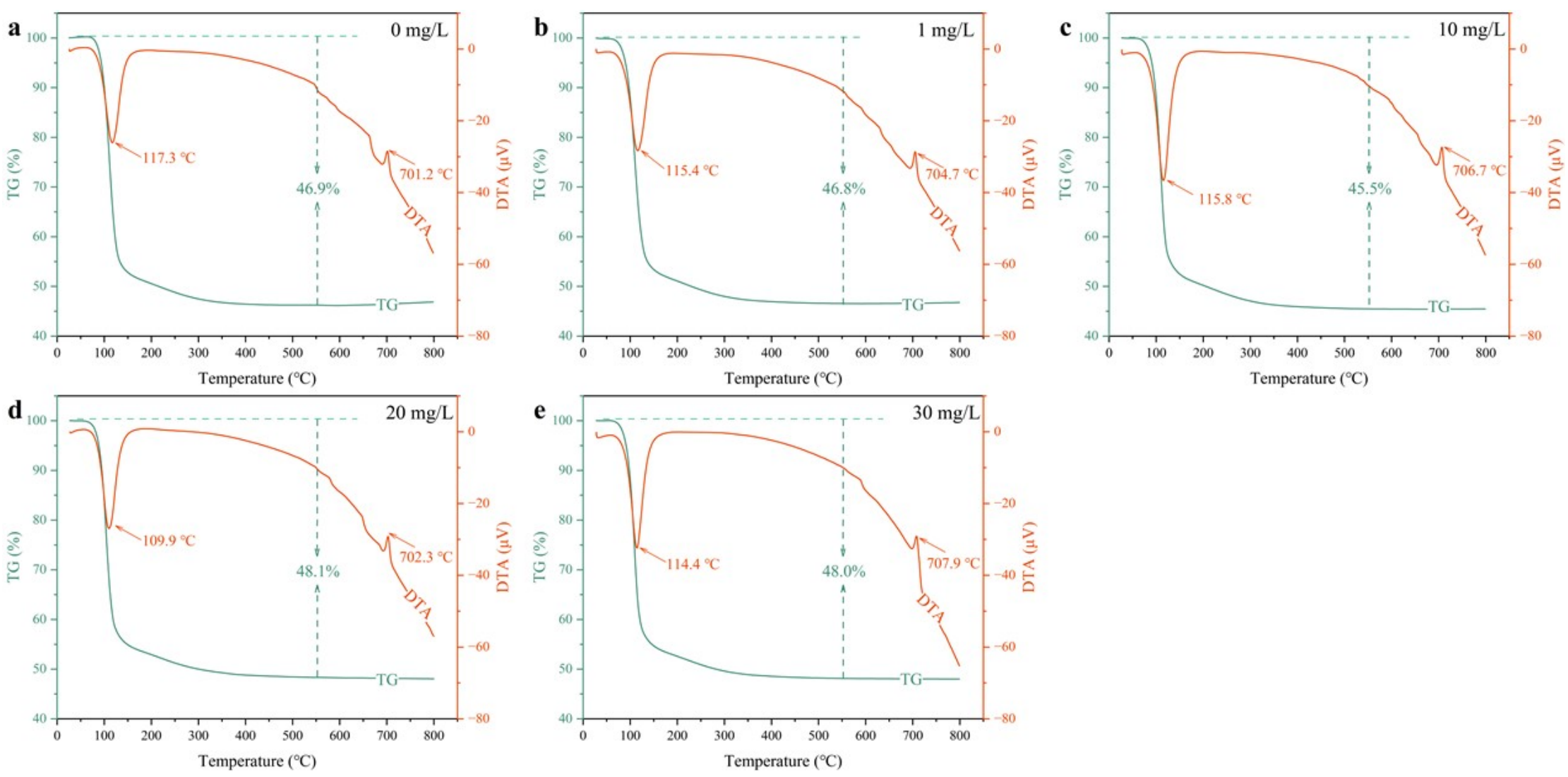

Fig. S3 Thermogravimetric analysis of struvite obtained by bacterial mineralization at Cd^{2+} concentrations of 0-30 mg/L (a-e). The substantial mass loss of struvite is mainly attributed to the release of six molecules of crystalline water and one ammonia molecule, corresponding to a strong endothermic peak at 109.9-117.3 ℃ in the DTA curve. The weak exothermic peak observed at 701.2-707.9 ℃ in the DTA curve suggests a possible transformation from an amorphous intermediate to crystalline γ-magnesium pyrophosphate ($Mg_2P_2O_7$) [1].

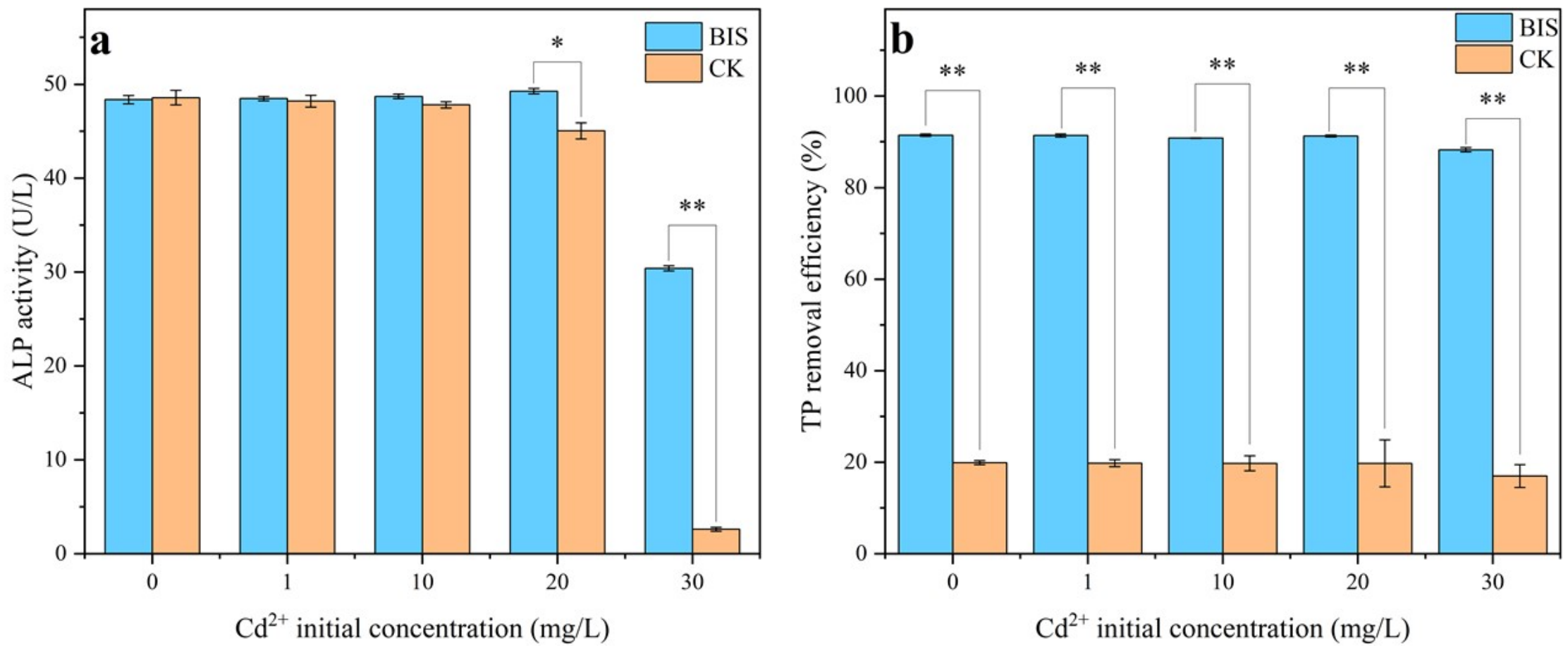

Fig. S4 Alkaline phosphatase (ALP) activity (a) and TP removal efficiency (b) in supernatants after 6 days incubation in the BIS and CK groups. "*" indicates $p < 0.05$, "**" indicates $p < 0.01$.

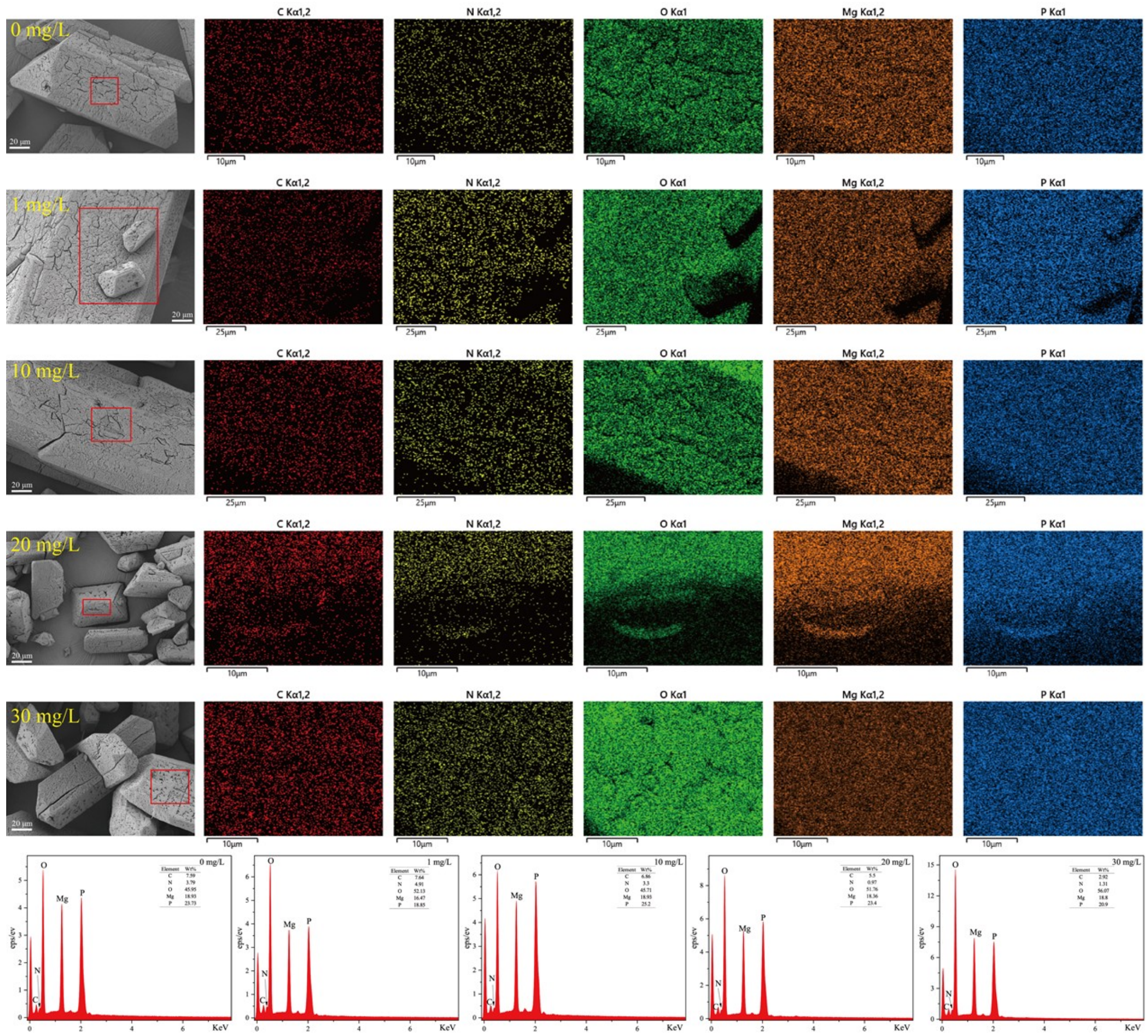

Fig. S5 EDS spectra of bio-struvite obtained at Cd^{2+} concentrations of 0-30 mg/L.

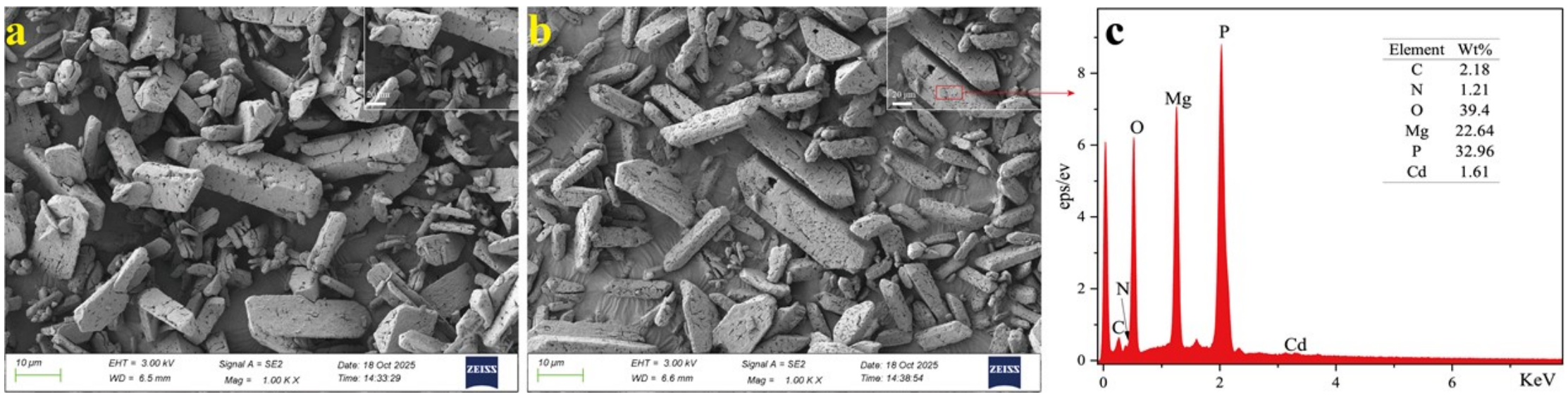

Fig. S6 Morphology and elemental composition of chem-struvite under different Cd^{2+} concentrations. (a, b) SEM images of chemically synthesized struvite at Cd^{2+} concentrations of 0-30 mg/L, respectively; (c) EDS spectrum of the crystal at 30 mg/L. Insets in each image show high-magnification views of the corresponding crystals.

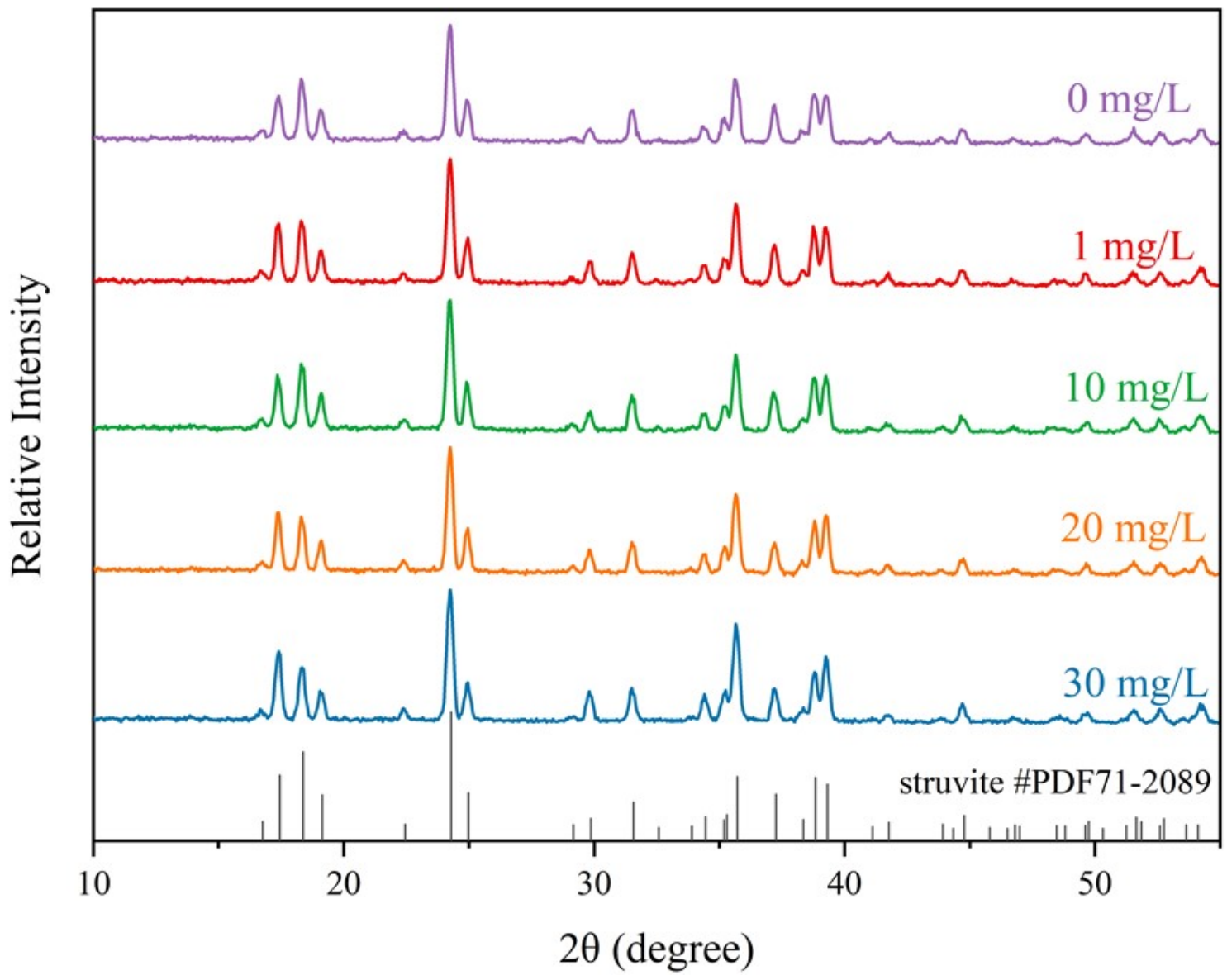

Fig. S7 XRD patterns of precipitates obtained by chemical precipitation at Cd^{2+} concentrations of 0-30 mg/L.

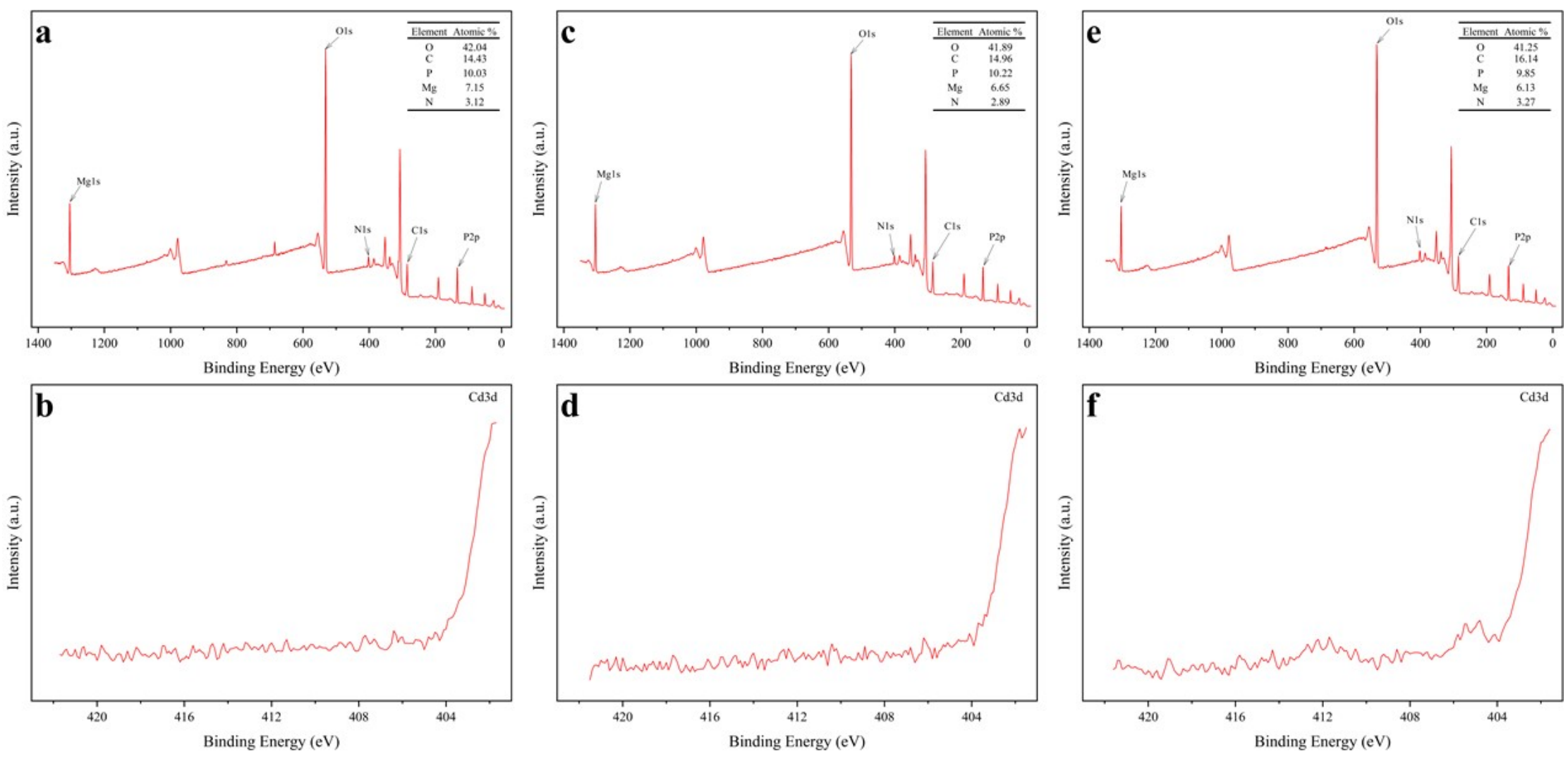

Fig. S8 XPS survey spectra and high-resolution Cd3d spectra of bacterially mineralized struvite under Cd^{2+} concentrations of (a, b) 1 mg/L, (c, d) 10 mg/L, and (e, f) 20 mg/L.

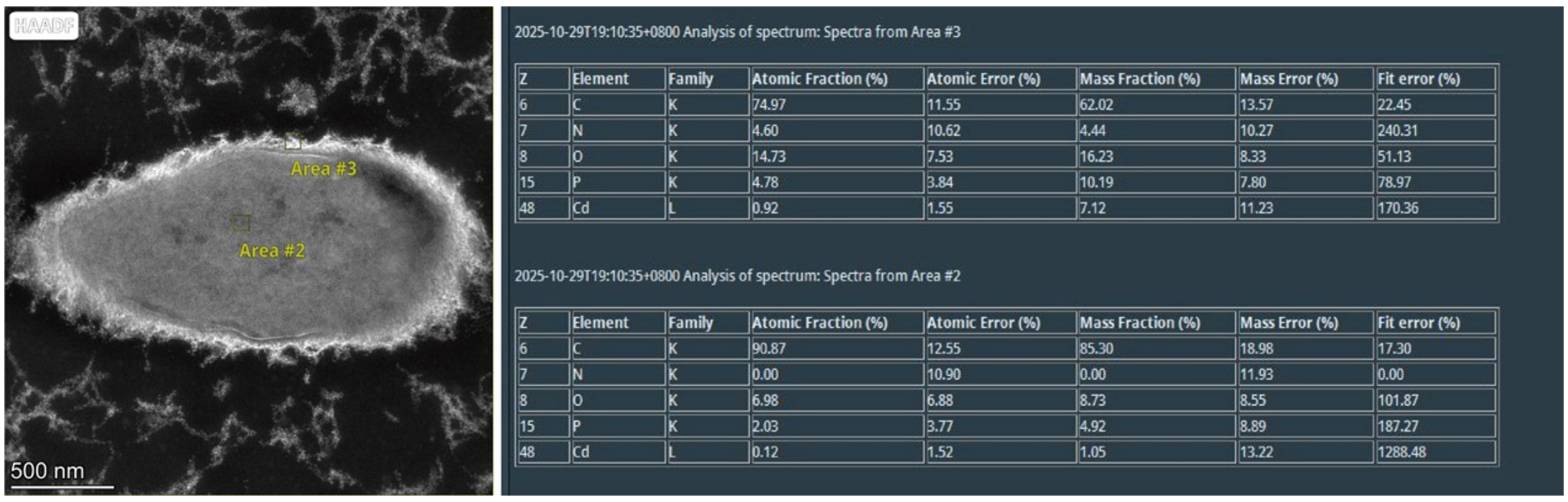

2025-10-29T19:10:35+0800 Analysis of spectrum: Spectra from Area #3

Z	Element	Family	Atomic Fraction (%)	Atomic Error (%)	Mass Fraction (%)	Mass Error (%)	Fit error (%)
6	C	K	74.97	11.55	62.02	13.57	22.45
7	N	K	4.60	10.62	4.44	10.27	240.31
8	O	K	14.73	7.53	16.23	8.33	51.13
15	P	K	4.78	3.84	10.19	7.80	78.97
48	Cd	L	0.92	1.55	7.12	11.23	170.36

2025-10-29T19:10:35+0800 Analysis of spectrum: Spectra from Area #2

Z	Element	Family	Atomic Fraction (%)	Atomic Error (%)	Mass Fraction (%)	Mass Error (%)	Fit error (%)
6	C	K	90.87	12.55	85.30	18.98	17.30
7	N	K	0.00	10.90	0.00	11.93	0.00
8	O	K	6.98	6.88	8.73	8.55	101.87
15	P	K	2.03	3.77	4.92	8.89	187.27
48	Cd	L	0.12	1.52	1.05	13.22	1288.48

Fig. S9 TEM-EDS point analysis results of MR-1 biosorbent after adsorption at Cd^{2+} concentration of 30 mg/L.

Tables

Table S1 Chemical composition of seawater.

Parameter	Values
pH	7.91
Salinity (‰)	24.62
Mg^{2+} (g/L)	1.11
Ca^{2+} (g/L)	0.34
K^+ (g/L)	0.41
Na^+ (g/L)	9.06
Cl^- (g/L)	14.27
SO_4^{2-} (g/L)	1.24

Note: The seawater was collected from the Yellow Sea off Yancheng City, Jiangsu Province, China (120.86°51′5.3″ E, 33.12°6′5.5″ N).

Table S2 Excitation and emission spectral distribution regions.

**Region**	**Excitation (nm)**	**Emission (nm)**	**Organic compounds**
I	200-250	250-330	tyrosine protein-like
II	200-250	330-380	tryptophan protein-like
III	200-250	>380	fulvic acid-like
IV	>250	<380	soluble microbial byproduct-like
V	>250	>380	humic acid-like

Table S3 Struvite content in precipitates obtained by bacterial mineralization at Cd^{2+} concentrations of 0-30 mg/L.

**Heavy metal ion**	**Concentration (mg/L)**	**Struvite content (%)**
Cd^{2+}	0	97.02 ± 1.13
Cd^{2+}	1	96.36 ± 0.06
Cd^{2+}	10	96.03 ± 0.32
Cd^{2+}	20	97.36 ± 2.34
Cd^{2+}	30	96.29 ± 2.15

Note: Values are presented as mean ± standard deviation (n = 3).

Table S4 Indicators related to struvite obtained by bacterial mineralization and chemical precipitation at Cd^{2+} concentrations of 0-30 mg/L.

	**Cd^{2+} concentration (mg/L)**	**Struvite yield (g/L)**	**TP removal efficiency (%)**	**TP recovery efficiency (%)**
biomineralization system	0	0.83 ± 0.0081 a	91.46 ± 0.2672 a	70.34 ± 0.6885 a
	1	0.82 ± 0.0136 a	91.41 ± 0.3394 a	69.55 ± 1.1544 a
	10	0.82 ± 0.0033 a	90.82 ± 0.0629 a	69.82 ± 0.2812 a
	20	0.83 ± 0.0085 a	91.30 ± 0.1900 a	70.64 ± 0.7220 a
	30	0.79 ± 0.0054 b	88.27 ± 0.2943 b	67.35 ± 0.4552 b
chemical precipitation system	0	0.70 ± 0.0019 a	60.23 ± 0.0543 a	59.54 ± 0.7414 a
	1	0.69 ± 0.0047 a	59.29 ± 0.1421 a	58.69 ± 0.2438 a
	10	0.66 ± 0.0101 b	60.02 ± 0.1832 a	56.14 ± 0.4725 b
	20	0.61 ± 0.0057 c	59.93 ± 0.2101 a	51.88 ± 0.9643 c
	30	0.58 ± 0.0068 d	58.12 ± 0.1416 a	49.33 ± 0.7412 d

Note: Different lowercase letters indicate significant differences among groups ($p < 0.05$); values are presented as mean ± standard deviation (n = 3).

Table S5 Langmuir and Freundlich adsorption isotherm parameters for Cd^{2+} adsorption by MR-1 biosorbent.

Biosorbents	**Langmuir**			**Freundlich**		
	q_{max} (mg/g)	K_L (L/mg)	R^2	K_F ((mg/g)(mg/L)^n)	n	R^2
MR-1 strain	28.02	0.2473	0.9976	9.2087	3.5600	0.9960

Table S6 Comparison of q_{max} for Cd^{2+} adsorption by biosorbent

**Biosorbent**	**q_{max} (mg/g)**	**References**
Bacillus cereus RC-1	24.01	2
Bacillus sp. B19	10.66	3
Escherichia coli	22.44	4
Bacillus subtilis	56.50	4
Saccharomyces cerevisiae	37.75	4
Shewanella oneidensis MR-1	28.02	This work

Table S7 Pseudo-first-order and pseudo-second-order kinetic equation parameters for Cd^{2+} adsorption by MR-1 biosorbent.

**Biosorbents**	**Pseudo-first-order**			**Pseudo-second-order**		
	q_e (mg/g)	K_1 (L/mg)	R^2	q_e (mg/g)	K_2 (g·mg^{-1}min^{-1})	R^2
MR-1 strain	26.58	1.5447	0.0431 1	26.58	0.02436	0.9973

Table S8 Changes in FTIR peak positions of MR-1 biosorbent before and after adsorption at a Cd^{2+} concentration of 30 mg/L.

**Before adsorption（cm^{-1}）**	**After adsorption（cm^{-1}）**	**Corresponding chemical bond/Functional group**	**Changes and possible reasons**
3297-3500	3298-3580	O-H/N stretching	Peak broadening indicates the involvement of hydroxyl and amino groups in Cd^{2+} coordination or changes in hydrogen bonding environments.
1543	1541	Amide II: N-H bending, C-N stretching	Minor peak shift suggests the participation of N-H and C-N groups in complexation with Cd^{2+}.
1454, 1400	1448, 1385	$-COO^-$ bending vibration	Peak shift indicates the involvement of carboxylate groups in surface complexation or ion exchange with Cd^{2+}.
1234	1236	P=O/P-O-C (phosphate esters, phospholipids, polysaccharides)	Slight rightward shift suggests phosphate groups participate in Cd^{2+} coordination or the formation of metal-phosphorus complexes.
1076	1020	Polysaccharide C-O, P-O, C-O-C vibrations (EPS/cell wall)	Pronounced displacement and shape change indicate the formation of new complex structures between Cd^{2+} and extracellular polysaccharides or phosphate groups, which are major adsorption sites.
619, 543	700, 573	Metal-ligand vibrations (e.g., Cd-O/Cd-P)	Significant peak shift suggests the possible formation of Cd-O or Cd-phosphate coordination/precipitation structures.

Reference

1 M.V. Ramlogan and A.A. Rouff, *J. Therm. Anal. Calorim*., 2015, **123**, 145-152.

2 F. Huang, Z. Dang, C. Guo, G. Lu, R.R. Gu, H. Liu and H. Zhang, *Colloids and Surfaces B: Biointerfaces*, 2013, **107**, 11-18.

3 S. Liu, Q. Pu, T. Mo, G. Peng, Y. Sun, Y. Zhang, J. Wang, Y. Li and H. Xu, *Water, Air, &Amp; Soil Pollution*, 2023, **234**, 543.

4 H. Huang, Q. Jia, W. Jing, H. Dahms and L. Wang, *Chemosphere*, 2020, **251**, 126428.
